# RhoE downregulation leads to enhanced cholesterol biosynthesis and sorafenib resistance in hepatocellular carcinoma

**DOI:** 10.1016/j.jbc.2025.110918

**Published:** 2025-11-11

**Authors:** Jingxiu Feng, Ling Ye, Changyuan Chen, Yiyi Zhu, Rumin Liu, Haolong Yu, Lijun Lu, Ke Gong, Wenhua Li

**Affiliations:** 1 Hubei Key Laboratory of Cell Homeostasis, College of Life Sciences, Wuhan University, Wuhan, China; 2 Wuhan University Shenzhen Research Institute, Shenzhen, China; 3 Hubei Province Key Laboratory of Allergy and Immunology, Taikang Medical School (School of Basic Medical Sciences), Taikang Center for Life and Medical Sciences, Wuhan University, Wuhan, China; 4 Department of Respiratory Medicine, Wuhan Hospital of Traditional Chinese Medicine, Wuhan, China

**Keywords:** HCC, sorafenib resistance, FAK, cholesterol, RhoE

## Abstract

Sorafenib remains the first-line systemic therapy for hepatocellular carcinoma (HCC), but its clinical efficacy is limited by acquired resistance. Here, we identified a significant association between acquired resistance to sorafenib and activation of the FAK signaling pathway. *In vitro* experiments confirmed that sorafenib suppresses Rho-related GTP-binding protein RhoE (RhoE) expression via Raf/MEK/ERK inhibition, thereby relieving its inhibitory effect on the transforming protein RhoA (RhoA) and Rho-associated protein kinase signaling pathway and ultimately leading to elevated phosphorylation of FAK at tyrosine 397 in HCC cells. Activated FAK subsequently promotes 3-hydroxy-3-methylglutaryl-coenzyme A reductase (HMGCR) expression *via* AKT signaling, thereby increasing intracellular cholesterol levels, which in turn upregulates glioma-associated oncogene homolog 1 (GLI1) expression and induces drug resistance. Genetic knockout of FAK or pharmacological inhibition using defactinib or PF-573228 effectively suppressed the sorafenib-induced upregulation of AKT and HMGCR. Notably, combination treatment with FAK inhibitors and sorafenib synergistically inhibited HCC cell viability, an effect reversed by HMGCR overexpression or exogenous cholesterol. *In vivo* and organoid experiments further demonstrated that combining sorafenib with defactinib significantly suppressed tumor growth and resistance signaling. Furthermore, bioinformatic analyses revealed that a gene signature related to cholesterol biosynthesis is significantly associated with poor prognosis in sorafenib-treated HCC patients, indicating its potential as a predictive biomarker. Collectively, this study identify systematically uncovers a new mechanism of sorafenib resistance in HCC: RhoE downregulation–mediated activation of the FAK/AKT–cholesterol–SHH/GLI1 axis as a key driver of sorafenib resistance and provide a rationale for combining FAK inhibitors with RAF-targeted therapies and for using cholesterol biosynthesis signatures to guide personalized treatment.

Primary liver cancer is the sixth most common malignancy worldwide and ranks third in cancer-related mortality. Among its subtypes, hepatocellular carcinoma (HCC) accounts for 75% to 85% of cases (1). Despite significant advancements in prevention and early detection, HCC remains a formidable clinical challenge. More than half of patients are diagnosed at an advanced stage, and approximately 70% experience disease recurrence within 5 years of treatment (2). The overall 5-year relative survival rate remains dismally low, at just 18% (3). For patients with intermediate to advanced HCC, systemic antitumor therapy plays a critical role in controlling disease progression and improving survival outcomes. Sorafenib, the first U.S. Food and Drug Administration-approved first-line systemic therapy for HCC has long served as the cornerstone of treatment (3). Sorafenib is a multikinase inhibitor (4) that exerts its antitumor effects through two primary mechanisms: it inhibits the Ras/Raf/MEK/ERK signaling pathway by targeting Raf-1 and B-Raf, thereby suppressing tumor cell proliferation, and it blocks angiogenesis by targeting receptor tyrosine kinases such as PDGFR-β and VEGFR (5). Although new therapeutic regimens—such as lenvatinib and the combination of atezolizumab with bevacizumab—have emerged in recent years (6), sorafenib continues to represent a fundamental treatment option. However, its clinical efficacy is significantly limited: only about 30% of patients show a treatment response, and most develop acquired resistance within 6 months (7). This therapeutic limitation underscores the urgent need for strategies that target resistance-associated mechanisms to enhance treatment effectiveness.

Focal adhesion kinase (FAK) is a nonreceptor tyrosine kinase that is frequently overexpressed in various solid tumors (8). Its autophosphorylation at Tyr397 promotes tumor cell migration, survival, and proliferation (9, 10). In HCC, FAK overexpression, and aberrant activation have been implicated in tumor progression and therapeutic resistance (11, 12, 13). Although several FAK inhibitors are currently under development, their efficacy as monotherapies has been limited in clinical trials (14, 15, 16, 17). However, accumulating evidence suggests that combination strategies hold significant promise. FAK inhibitors have been shown to enhance the efficacy of chemotherapy and targeted therapies and to improve responses to immunotherapy (18). Several combination regimens incorporating FAK inhibitors have already advanced to phase II clinical trials (19, 20, 21, 22), highlighting their translational potential. RhoE, as a downstream effector of ERK, can suppress the activation of ROCK, while ROCK activation has been reported to be closely associated with FAK activation. Therefore, the crosstalk between RhoE and FAK may contribute to sorafenib resistance in HCC and provide a novel potential target for overcoming this resistance.

In this study, we found that short-term sorafenib treatment induces upregulation of FAK phosphorylation at Tyr397 in HCC cells, with persistently elevated levels observed in resistant cell lines. Mechanistic investigations revealed that sorafenib increases FAK phosphorylation by downregulating RhoE expression, thereby activating the RHOA/ROCK signaling pathway. Subsequently, the FAK/AKT signaling axis upregulates HMGCR expression to enhance cholesterol biosynthesis, thereby increasing glioma-associated oncogene homolog 1 (GLI1) expression and inducing sorafenib resistance. Notably, the clinical-stage FAK inhibitor defactinib, when combined with sorafenib, exhibited significant synergistic effects in HCC cell lines, HCC organoids, and cell line–derived xenograft models. These findings underscore the critical role of compensatory FAK signaling activation in mediating sorafenib resistance and provide a strong rationale for optimizing combination therapy strategies in HCC. Targeting FAK-driven compensatory pathways may represent a promising therapeutic approach to overcoming sorafenib resistance and improving clinical outcomes for patients with advanced HCC.

## Results

### FAK activation mediates adaptive resistance to sorafenib in HCC

**Figure 1:**
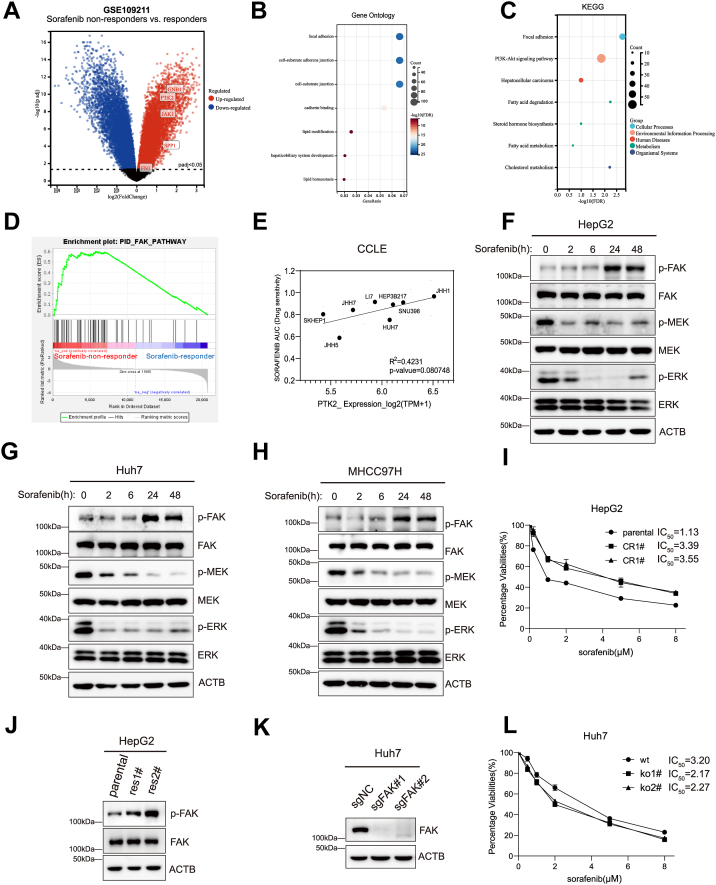
**Identifying FAK as a therapeutic target for overcoming sorafenib resistance in HCC**. *A*, volcano plot depicting differentially expressed genes between sorafenib nonresponders and responders. The *horizontal dashed line* represents a q-value threshold of 0.05. Differentially expressed genes were identified using DESeq2, with *red dots* indicating upregulated genes in nonresponders and *blue dots* representing downregulated genes. *B* and *C*, Kyoto Encyclopedia of Genes and Genomes and Gene Ontology enrichment analyses of upregulated genes in sorafenib nonresponders. The bubble size corresponds to the number of enriched genes, while the *x*-axis represents the adjusted *p* value. The color gradient of bubbles indicates the statistical significance level (adjusted *p* value). *D*, gene set enrichment analysis of the “PID_FAK_PATHWAY” gene set in the phase 3 STORM trial cohort, comprising 46 sorafenib nonresponders and 21 responders. The *green line* represents the Enrichment Score, and the *black vertical lines* denote genes belonging to the analyzed gene set. *E*, scatter plot demonstrating the correlation between sorafenib area under the curve values and *PTK2* expression levels in HCC cell lines, indicating a potential relationship between *PTK2* expression and sorafenib sensitivity. *F–H*, time-dependent changes in p-FAK, FAK, p-MEK, MEK, p-ERK, and ERK protein levels were examined by Western blotting following treatment of HepG2 (3 μM), Huh7 (2 μM), and MHCC97H (5 μM) cells with sorafenib. *I*, parental HepG2 cells and two sorafenib-resistant HepG2 cell lines were treated with varying concentrations of sorafenib for 72 h, and cell viability was assessed using Alamar Blue assay. IC_50_ values were calculated using GraphPad Prism. *J*, Western blot analysis of p-FAK and FAK protein expression in parental HepG2 cells and two sorafenib-resistant HepG2 cell lines. *K*, Western blot analysis showing FAK protein levels in two FAK-KO Huh7 cell lines and WT Huh7 cells. *L*, WT Huh7 cells and two FAK-KO cell lines were treated with varying concentrations of sorafenib for 72 h, and cell viability was measured using Alamar Blue assay. IC_50_ values were determined using GraphPad Prism. Statistical significance for panel was assessed using Pearson correlation analysis with linear regression; R^2^ and *p* value are shown in the plot. FAK, focal adhesion kinase; HCC, hepatocellular carcinoma; PTK2, protein tyrosine kinase 2.

To investigate whether FAK is involved in the resistance mechanism of HCC to sorafenib, we first analyzed clinical data. Transcriptomic data from a phase III clinical trial (23), which included 46 patients resistant to sorafenib and 21 patients who responded effectively, were examined. Differential gene expression analysis revealed that multiple genes were significantly upregulated in sorafenib-resistant patients, including protein tyrosine kinase 2 (*PTK2)*, which encodes FAK (Fig. 1*A*). To further validate the involvement of the FAK pathway in sorafenib resistance, we performed Kyoto Encyclopedia of Genes and Genomes and Gene Ontology pathway enrichment analyses, which demonstrated significant enrichment of the FAK signaling pathway in resistant patients (Fig. 1, *B* and *C*). Additionally, Gene Set Enrichment Analysis confirmed an upregulation trend of FAK pathway–related genes in these patients (Fig. 1*D*), suggesting that the FAK pathway is markedly activated in sorafenib-resistant compared to sorafenib-sensitive patients. Previous studies have shown that FAK overexpression and hyperactivation in HCC are associated with amplification of the *PTK2* gene (24). Whole-genome sequencing data indicate that approximately 26.1% of HCC patients harbor *PTK2* amplification (25). Chromosomal fragment analysis in HCC samples revealed significant aberrant amplification of the 8q24 region, where *PTK2* is located (Fig. S1*A*). Further gene set enrichment analysis based on clinical trial transcriptomic data demonstrated that the “CHR8Q24” gene set was significantly enriched in sorafenib-resistant patients (Fig. S1*B*), suggesting that 8q24 amplification may contribute to sorafenib resistance by driving FAK overexpression and hyperactivation. To assess the broader relevance of these findings, we conducted a large-scale analysis using The Cancer Genome Atlas database. Results showed that 57.8% of HCC patients exhibited FAK copy number amplification (Fig. S1*C*), and that FAK expression levels were significantly higher in tumor tissues than in normal liver tissues (Fig. S1*D*). Moreover, analysis of the Cancer Cell Line Encyclopedia (CCLE) database revealed a positive correlation between FAK expression levels and sorafenib resistance in HCC cell lines (Fig. 1*E*).

Based on bioinformatics analyses suggesting a potential association between FAK and sorafenib resistance, we conducted experimental validation to further investigate this hypothesis. First, we examined whether sorafenib treatment induces upregulation of phosphorylated FAK (p-FAK). In HepG2, Huh7, and MHCC97H cells, sorafenib effectively suppressed p-MEK and p-ERK levels; however, p-FAK levels increased markedly at 24 h and 48 h (Fig. 1, *F*–*H*). To further assess the long-term effects of sorafenib on p-FAK levels, we established two sorafenib-resistant HepG2 cell lines by continuously treating cells with sorafenib for 2 months. IC_50_ assays revealed that the parental HepG2 cells had an IC_50_ of 1.13 μM, whereas the two resistant lines exhibited IC_50_ values exceeding 3.3 μM (Fig. 1*I*). Notably, p-FAK levels were significantly elevated in the resistant cell lines compared to parental cells (Fig. 1*J*), consistent with findings from the short-term treatment experiments. These results indicate that sorafenib induces compensatory upregulation of p-FAK in HCC cells. To further validate the role of FAK in sorafenib resistance in HCC, we generated two FAK-KO Huh7 cell lines (Fig. 1*K*). Cell viability assays showed that, compared with WT Huh7 cells, FAK-KO cells exhibited significantly increased sensitivity to sorafenib (Fig. 1*L*). These results suggest that sorafenib-treated HCC cells may acquire resistance through the upregulation of p-FAK levels.

### Pharmacologic FAK blockade synergizes with sorafenib to overcome adaptive resistance in HCC

In our previous work, analysis of sorafenib resistance–associated genes and copy number variations in HCC suggested that the FAK signaling pathway may be involved in sorafenib resistance. Subsequent validation experiments in HCC cell lines revealed that p-FAK levels were significantly upregulated following either short-term sorafenib treatment or in long-term sorafenib-resistant cell lines. Notably, knockout of the FAK gene markedly enhanced the sensitivity of HCC cells to sorafenib. These findings suggest that adaptive activation of FAK may represent a key mechanism underlying the acquisition of sorafenib resistance in HCC.

**Figure 2:**
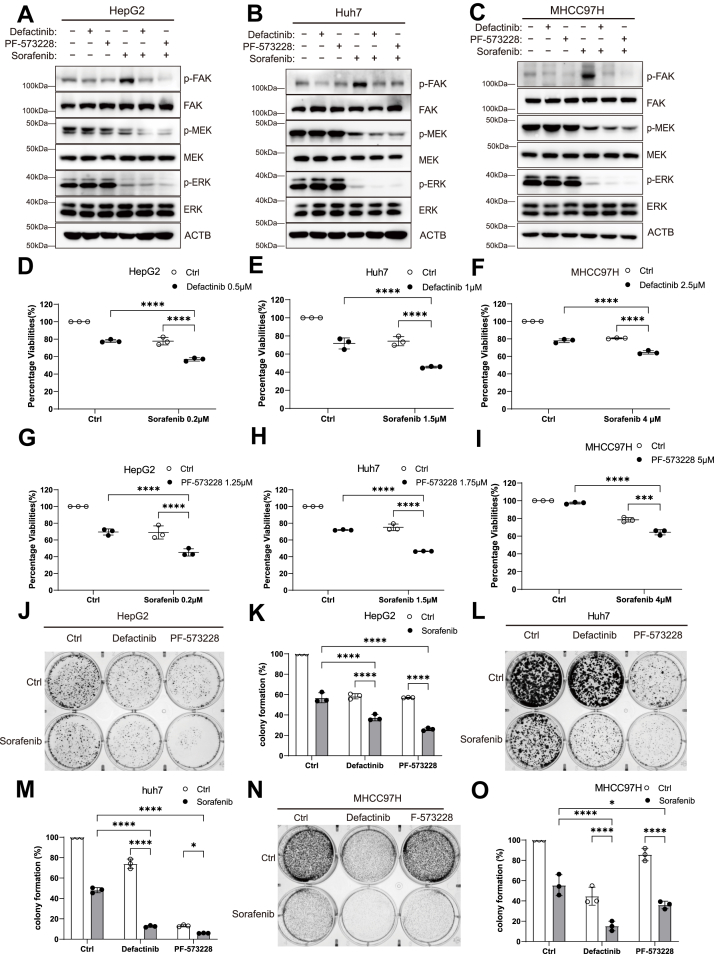
**FAK inhibitor in combination with sorafenib blocks FAK activation-induced resistance and synergistically enhances tumor cell killing**. *A–C*, protein levels of p-FAK, FAK, p-MEK, MEK, p-ERK, and ERK were analyzed by Western blotting following 24 h of treatment with sorafenib and the FAK inhibitors defactinib and PF-573228 in HepG2, Huh7, and MHCC97H cells. *D–I*, cell viability was measured using the Alamar Blue assay following 72 h of treatment with sorafenib and the FAK inhibitors defactinib and PF-573228 in HepG2, Huh7, and MHCC97H cells. *J*, colony formation assay in HepG2 cells treated for 14 days with 1.25 μM defactinib and 1.5 μM PF-573228 in combination with 1 μM sorafenib. *K*, quantification of colony formation in HepG2 cells following combination treatment. *L*, colony formation assay in Huh7 cells treated for 14 days with 1.5 μM defactinib and 1.5 μM PF-573228 in combination with 2 μM sorafenib. *M*, quantification of colony formation in Huh7 cells following combination treatment. *N*, colony formation assay in MHCC97H cells treated for 14 days with 3 μM defactinib and 4 μM PF-573228 in combination with 3 μM sorafenib. *O*, quantification of colony formation in MHCC97H cells following combination treatment. Statistical significance for *panels D*, *E*, *F*, *G*, *H*, *I*, *K*, *M*, *and O* was determined using two-way ANOVA followed by Bonferroni’s *post hoc* test. Data are presented as mean ± SD from at least three independent experiments. Significance is indicated as *p* ≥ 0.05 (ns), *p* < 0.05 (∗), *p* < 0.01 (∗∗), *p* < 0.001 (∗∗∗), and *p* < 0.0001 (∗∗∗∗). FAK, focal adhesion kinase; p-FAK, phosphorylated FAK.

Based on these observations, we further investigated whether FAK inhibition could mitigate this resistance. As an initial step, we tested whether FAK inhibitors could suppress the sorafenib-induced increase in p-FAK. HepG2, Huh7, and MHCC97H cells were cotreated with sorafenib and two small-molecule FAK inhibitors—defactinib and PF-573228. Western blot analysis demonstrated that sorafenib-induced p-FAK upregulation was effectively suppressed by both inhibitors (Fig. 2, *A*–*C*), confirming that FAK inhibition counteracts this adaptive response. Next, we evaluated the impact of combining FAK inhibitors with sorafenib on HCC cell viability. Cells were treated with sorafenib plus either defactinib or PF-573228 for 72 h, followed by cell viability assays. The combination therapy exhibited significantly greater growth-inhibitory effects than either agent alone (Fig. 2, *D*–*I*). To assess long-term effects, a 10-day colony formation assay was performed, yielding consistent results—the combination treatment markedly suppressed colony formation compared to treatment with sorafenib or FAK inhibitors alone (Fig. 2, *J*–*O*).

Collectively, our findings demonstrate that HCC cells treated with sorafenib undergo adaptive FAK activation, which contributes to resistance. Importantly, coinhibition of FAK and RAF/MEK/ERK signaling produces a synergistic antitumor effect, highlighting FAK inhibition as a promising strategy to overcome sorafenib resistance in HCC.

### Sorafenib-mediated MAPK inhibition suppresses RhoE, unleashing the RhoA/ROCK/FAK adaptive loop in HCC cells

**Figure 3:**
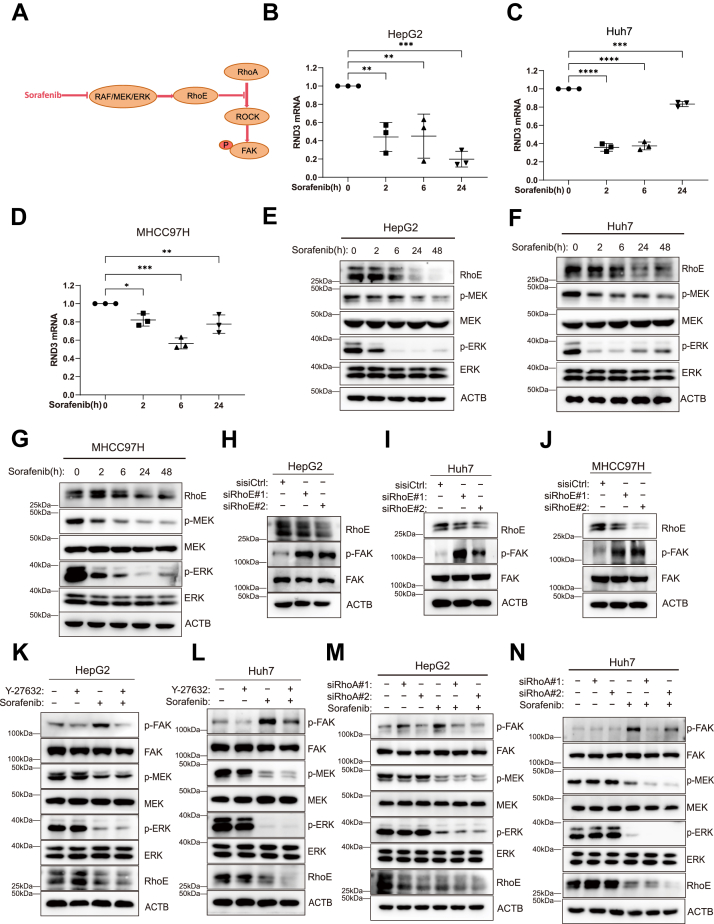
**Sorafenib downregulates RhoE *via* MAPK blockade, activating the RhoA/ROCK/FAK compensatory loop in HCC**. *A*, sorafenib treatment suppresses RhoE expression, thereby relieving its inhibitory effect on the RhoA/ROCK pathway. This leads to increased p-FAK. *B–D*, RhoE mRNA levels were quantified by qPCR after treatment of HepG2 (3 μM), Huh7 (2 μM), and MHCC97H (5 μM) cells with sorafenib. *E–G*, time-dependent changes in RhoE, p-MEK, MEK, p-ERK, and ERK protein levels were analyzed by Western blotting following sorafenib treatment of HepG2, Huh7, and MHCC97H cells. *H–J*, following RhoE knockdown with two distinct siRNAs, RhoE, p-FAK, and FAK protein levels were assessed by Western blotting. *K* and *L*, HepG2 and Huh7 cells were treated for 24 h with sorafenib and the ROCK inhibitor Y-27632. Protein levels of p-FAK, FAK, p-MEK, MEK, p-ERK, ERK, and RhoE were then analyzed by Western blotting. *M* and *N*, following RhoA knockdown using two distinct siRNAs, cells were treated with sorafenib, and changes in p-FAK, FAK, p-MEK, MEK, p-ERK, ERK, and RhoE protein levels were assessed by Western blotting. Statistical significance for *panels B–D* was determined using one-way ANOVA followed by Bonferroni’s *post hoc* test. Data are presented as mean ± SD from at least three independent experiments. Significance is indicated as *p* ≥ 0.05 (ns), *p* < 0.05 (∗), *p* < 0.01 (∗∗), *p* < 0.001 (∗∗∗), and *p* < 0.0001 (∗∗∗∗). ROCK, Rho-associated protein kinase; FAK, focal adhesion kinase; p-FAK, phosphorylated FAK.

In our previous experiments, we observed that sorafenib treatment upregulates p-FAK in HCC cells, thereby contributing to sorafenib resistance. However, combining sorafenib with FAK inhibitors effectively overcomes this resistance. To investigate the mechanism underlying the upregulation of p-FAK in HCC cells following sorafenib treatment, we focused on the canonical RhoA/ROCK axis, which is widely implicated in FAK activation (26, 27, 28); Notably, RhoE competitively binds to ROCK in place of RhoA, and the interaction between RhoE and ROCK inhibits ROCK activation. Recent studies have shown that the Raf/MEK/ERK pathway inhibitor avutometinib can downregulate RhoE (encoded by RND3), a downstream effector of MAPK signaling, thereby relieving RhoE-mediated inhibition of the RhoA/ROCK pathway and leading to increased phosphorylation of FAK. (29). Therefore, we hypothesized that sorafenib upregulates p-FAK by activating the RhoA/ROCK pathway *via* suppression of RhoE (Fig. 3*A*). To test this hypothesis, we first performed quantitative polymerase chain reaction (qPCR) analysis, which revealed that sorafenib significantly downregulated *RND3* mRNA in HepG2, Huh7, and MHCC97H cells (Fig. 3, *B*–*D*). Consistently, Western blotting showed that sorafenib reduced p-MEK, p-ERK, and RhoE protein levels at both 24 h and 48 h (Fig. 3, *E*–*G*), indicating that this effect is mediated through inhibition of the Raf/MEK/ERK pathway. To determine whether RhoE loss is sufficient to activate FAK, we knocked down RhoE using siRNA in all three cell lines, which led to a pronounced increase in p-FAK levels (Fig. 3, *H*–*J*). To further verify whether RhoE regulates p-FAK through the RhoA/ROCK pathway, we blocked this axis using either a ROCK inhibitor or RhoA-targeting siRNA. Western blot analysis showed that the sorafenib-induced increase in p-FAK was reversed by the ROCK inhibitor Y-27632 (Fig. 3, *K* and *L*), and siRNA-mediated knockdown of RhoA similarly abrogated p-FAK upregulation (Fig. 3, *M* and *N*; Fig. S1, *E* and *F*), confirming that the RhoA/ROCK axis is essential in this process.

Taken together, our data indicate that sorafenib suppresses Raf/MEK/ERK signaling, thereby downregulating RhoE, relieving inhibition of the RhoA/ROCK axis, and ultimately triggering compensatory upregulation of p-FAK. This signaling cascade provides a mechanistic explanation for the development of sorafenib resistance in HCC and suggests that concurrent blockade of the RhoA/ROCK/FAK pathway could be a viable strategy to enhance therapeutic efficacy.

### Targeting FAK inhibits sorafenib-induced cholesterol biosynthesis

**Figure 4:**
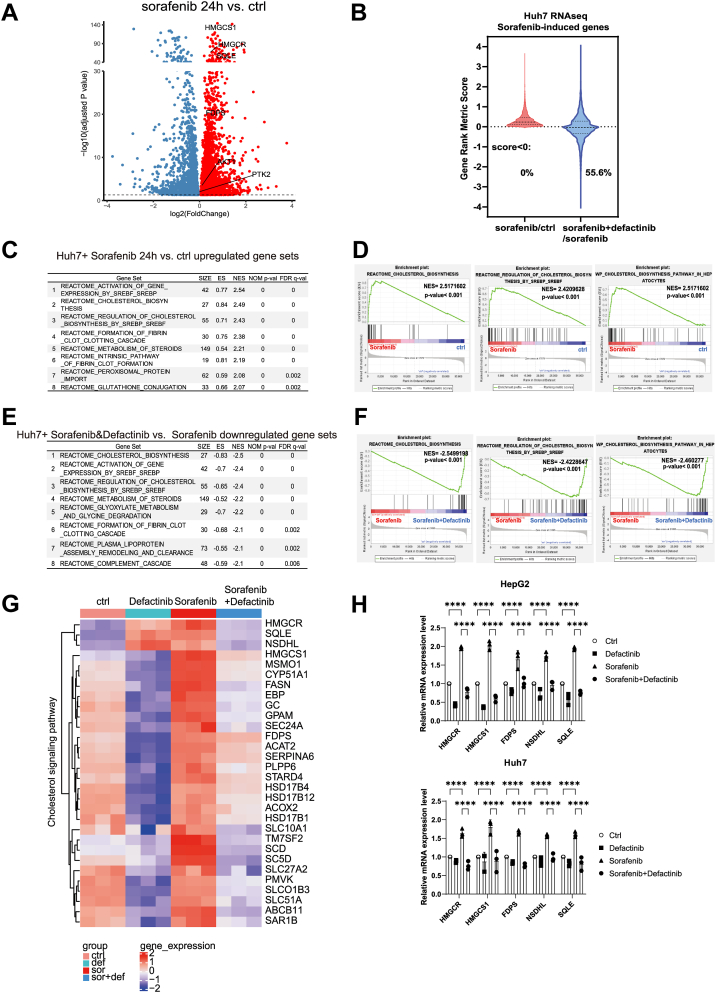
**Sorafenib-induced cholesterol signaling pathway can be blocked by FAK inhibitor**. *A*, volcano plot depicting differentially expressed genes between Huh7 cells treated with sorafenib for 24 h and untreated control cells. The *horizontal dashed line* represents a *p* value threshold of 0.05, and differentially expressed genes were identified using DESeq2. *Red dots* indicate upregulated genes in sorafenib-treated Huh7 cells, while *blue dots* represent downregulated genes. *B*, violin plot illustrating genes with z-normalized gene set enrichment analysis gene rank metric scores >0 in the sorafenib *versus* control comparison. A total of 55.6% of sorafenib-upregulated genes were suppressed by defactinib, indicating a potential role of FAK inhibition in reversing sorafenib-induced transcriptional changes. *C–F*, tables summarizing the top eight enriched signaling pathways in the sorafenib *versus* control and sorafenib+defactinib *versus* sorafenib comparisons. Gene set enrichment analysis snapshots highlight the enrichment of cholesterol-related signaling pathways in both comparisons. The *green line* represents the Enrichment Score, while *black vertical lines* denote genes within the examined gene set. *G*, heat map displaying the expression of cholesterol signaling pathway genes across the control, defactinib, sorafenib, and sorafenib+defactinib treatment groups, illustrating the impact of FAK inhibition on sorafenib-induced cholesterol pathway activation. *H*, qPCR analysis of HMGCR, HMGCS1, FDPS, NSDHL, and SQLE mRNA expression in HepG2 and Huh7 cells following 24-h cotreatment with defactinib, PF-573228, and sorafenib. HepG2 cells were treated with 2 μM defactinib, 3 μM PF-573228, and 1 μM sorafenib, while Huh7 cells were treated with 3 μM defactinib, 4 μM PF-573228, and 2 μM sorafenib. Statistical significance for *panels D and F* was assessed using the GSEA algorithm (Broad Institute) with 1000 gene set permutations. Normalized enrichment score (NES) and nominal *p* value are shown. Statistical significance for *panels H* was determined using two-way ANOVA followed by Bonferroni’s *post hoc* test. Data are presented as mean ± SD from at least three independent experiments. Significance is indicated as *p* ≥ 0.05 (ns), *p* < 0.05 (∗), *p* < 0.01 (∗∗), *p* < 0.001 (∗∗∗), and *p* < 0.0001 (∗∗∗∗). FAK, focal adhesion kinase; HMGCR, 3-hydroxy-3-methylglutaryl-coenzyme A reductase.

Building on our previous findings that p-FAK upregulation mediates sorafenib resistance in HCC, we further investigated the downstream molecular mechanisms by which FAK activation contributes to drug resistance. To this end, we performed RNA-Seq on Huh7 cells treated with sorafenib alone or in combination with defactinib. Differential gene expression analysis revealed that after 24 h of sorafenib monotherapy, numerous genes were significantly upregulated, including HMGCR, HMGCS1, and SQLE (Fig. 4*A*), which are known to be involved in cholesterol biosynthesis and potentially in the development of drug resistance. Further gene set enrichment analysis showed that 55.6% of the genes upregulated in the sorafenib group were significantly downregulated upon combination treatment (Fig. 4*B*), indicating that FAK inhibition suppresses sorafenib-induced resistance–associated transcriptional changes. To elucidate the underlying resistance mechanism, we focused on eight signaling pathways significantly enriched in the sorafenib monotherapy group (Fig. 4*C*). Notably, pathways related to cholesterol biosynthesis were highly enriched (Fig. 4*D*) and were markedly downregulated in the combination treatment group (Fig. 4, *E* and *F*).

Previous studies have reported that elevated cholesterol biosynthesis or hypercholesterolemia is closely associated with sorafenib resistance (30), supporting the hypothesis that FAK activation promotes resistance *via* dysregulation of cholesterol metabolism. Conversely, FAK inhibition may reverse this resistance by modulating cholesterol-related pathways. To identify key genes involved in cholesterol-mediated resistance, we performed a heat map analysis of cholesterol biosynthesis genes. The results showed widespread upregulation in the sorafenib group, while these genes were significantly suppressed upon combination treatment. In particular, HMGCR, HMGCS1, FDPS, NSDHL, and SQLE were prominently induced by sorafenib and downregulated by combination therapy (Fig. 4*G*). To validate the RNA-Seq findings, we conducted qPCR analysis of these key genes in HepG2 and Huh7 cells. Consistent with the sequencing data, HMGCR, HMGCS1, FDPS, NSDHL, and SQLE were significantly upregulated following sorafenib treatment and suppressed by cotreatment with defactinib (Fig. 4*H*).

In addition to the cholesterol pathway, we also examined other lipid metabolism–related pathways. RNA-Seq analysis revealed that fatty acid metabolism and triglyceride synthesis pathways were upregulated following sorafenib treatment (Fig. S2, *A* and *B*). Further validation showed that only the fatty acid metabolism pathway was significantly downregulated in the combination treatment group (Fig. S2, *C* and *D*), while the changes in phospholipid metabolism—upregulation in the sorafenib group and downregulation in the combination group—were not significant (Fig. S2, *E* and *F*). Accordingly, we selected three representative genes from each pathway (fatty acid metabolism: FASN, SLC25A20, ACLY; triglyceride synthesis: DGAT2, LPIN1, MOGAT3) for qPCR validation. However, in both HepG2 and Huh7 cells, time-course treatment with sorafenib did not result in significant upregulation of the mRNA levels of these genes (Fig. S2, *G*–*R*). Given the lack of consistent and sustained activation of these pathways, we redirected our focus to the cholesterol biosynthesis pathway.

In summary, these findings suggest that cholesterol metabolism plays a pivotal role in FAK-dependent sorafenib resistance in HCC cells, providing a mechanistic link between FAK signaling and therapeutic resistance.

### FAK-driven cholesterol biosynthesis mediates sorafenib resistance in HCC

**Figure 5:**
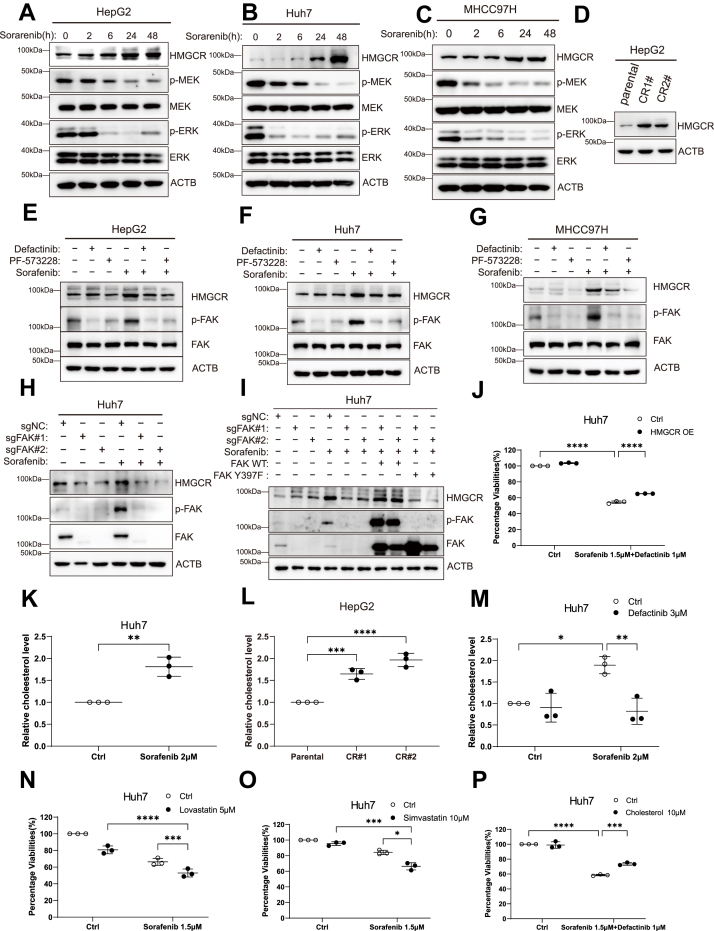
**Activation of the cholesterol synthesis pathway in HCC cells mediates drug resistance after sorafenib treatment**. *A–C*, protein levels of HMGCR, p-MEK, MEK, p-ERK, and ERK were analyzed by Western blotting following sorafenib treatment in HepG2, Huh7, and MHCC97H cells. *D*, Western blot analysis of HMGCR protein expression in parental HepG2 cells and two sorafenib-resistant HepG2 cell lines. *E–G*, following 24-h cotreatment with sorafenib and the FAK inhibitors defactinib and PF-573228, HMGCR, p-FAK, and FAK protein levels were analyzed by Western blotting in HepG2, Huh7, and MHCC97H cells. *H*, FAK, p-FAK, and HMGCR protein levels were examined by Western blotting in WT and FAK-KO Huh7 cells following 24-h sorafenib treatment. *I*, either WT FAK or the Y397F mutant was reintroduced into FAK-KO Huh7 cells, followed by 24-h sorafenib treatment. HMGCR, FAK, and p-FAK protein levels were analyzed by Western blotting. *J*, Huh7 cells overexpressing HMGCR were cotreated with defactinib and sorafenib for 48 h, and cell viability was assessed using the Alamar Blue assay. *K*, cellular cholesterol levels in Huh7 cells were measured using a cholesterol assay kit under basal conditions. Data are presented as mean ± SD (n = 3). *L*, cholesterol levels in parental HepG2 and two sorafenib-resistant HepG2 cell lines were measured using a cholesterol assay kit. *M*, cellular cholesterol levels in Huh7 cells were measured using a cholesterol assay kit following 24-h co-treatment with defactinib and sorafenib. *N* and *O*, cell viability in Huh7 cells was measured using the Alamar Blue assay following 72-h cotreatment with simvastatin or lovastatin in combination with sorafenib. *P*, cell viability in Huh7 cells was measured using the Alamar Blue assay following 72-h cotreatment with exogenous cholesterol and the combination of defactinib and sorafenib. Statistical significance for *panel K* was determined using unpaired two-tailed Student’s *t* test. Statistical significance for *panels J* and *L–P* was determined using two-way ANOVA followed by Bonferroni’s *post hoc* test. Data are presented as mean ± SD from at least three independent experiments. Significance is indicated as *p* ≥ 0.05 (ns), *p* < 0.05 (∗), *p* < 0.01 (∗∗), *p* < 0.001 (∗∗∗), and *p* < 0.0001 (∗∗∗∗). FAK, focal adhesion kinase; p-FAK, phosphorylated FAK; HMGCR, 3-hydroxy-3-methylglutaryl-coenzyme A reductase.

To validate these findings at the protein level, we performed Western blot analysis. In HepG2, Huh7, and MHCC97H cells, sorafenib treatment for 24 h and 48 h significantly increased HMGCR protein levels (Fig. 5, *A*–*C*). Moreover, in two sorafenib-resistant HepG2 cell lines, HMGCR was also significantly upregulated (Fig. 5*D*). These results suggest that aberrant upregulation of HMGCR in the cholesterol biosynthetic pathway may contribute to sorafenib resistance in HCC. Next, we evaluated the effect of FAK inhibition on HMGCR expression. The results showed that sorafenib significantly upregulated both p-FAK and HMGCR, whereas cotreatment with a FAK inhibitor effectively suppressed this upregulation (Fig. 5, *E*–*G*), indicating that HMGCR upregulation can be blocked by FAK inhibition. To further confirm FAK dependency, we compared WT and FAK-KO Huh7 cells. Sorafenib markedly increased p-FAK and HMGCR only in WT cells; this effect was absent in FAK-KO cells (Fig. 5*H*). Re-expression of WT FAK restored the sorafenib-induced upregulation of HMGCR, whereas the phosphorylation-deficient Y397F mutant failed to do so (Fig. 5*I*), further validating that HMGCR upregulation relies on FAK phosphorylation activity. Additionally, cell viability assays showed that forced overexpression of HMGCR partially rescued the growth inhibition caused by defactinib plus sorafenib treatment in Huh7 cells (Figs. 5*J*; Fig. S1*G*), supporting the notion that HMGCR functions downstream of FAK to promote drug resistance.

Based on these findings, we further examined the impact of sorafenib treatment on intracellular cholesterol levels in HCC cells. Using a total cholesterol assay kit, we found that intracellular cholesterol levels were significantly elevated in Huh7 cells following sorafenib treatment (Fig. 5*K*). In addition, sorafenib-resistant HepG2 cell lines exhibited significantly higher intracellular cholesterol levels than parental cells (Fig. 5*L*). Furthermore, sorafenib-induced cholesterol accumulation was markedly suppressed by combined treatment with the FAK inhibitor defactinib (Fig. 5*M*). These results suggest that sorafenib promotes intracellular cholesterol accumulation through FAK activation, contributing to drug resistance. Given that increased intracellular cholesterol may drive resistance, we investigated whether inhibition of cholesterol biosynthesis could enhance HCC cell sensitivity to sorafenib. We selected two cholesterol biosynthesis inhibitors (lovastatin and simvastatin) and performed cell viability assays. The results demonstrated that cotreatment with statins and sorafenib significantly reduced Huh7 cell viability, exhibiting greater efficacy than either agent alone (Fig. 5, *N* and *O*). To further validate the role of cholesterol metabolism in FAK-mediated sorafenib resistance, we conducted rescue experiments. Exogenous cholesterol supplementation reversed the inhibitory effects of sorafenib plus defactinib on Huh7 cell viability (Fig. 5*P*), further confirming that FAK activation contributes to resistance *via* upregulation of cholesterol biosynthesis.

Altogether, sorafenib treatment activates FAK, which subsequently upregulates the cholesterol biosynthesis pathway—particularly HMGCR—leading to increased intracellular cholesterol levels and drug resistance. Importantly, inhibition of the FAK–cholesterol biosynthesis axis significantly enhances the cytotoxic efficacy of sorafenib in HCC cells.

### GLI1 of the SHH pathway is a key downstream effector in cholesterol-mediated sorafenib resistance

To investigate the mechanism by which cholesterol mediates sorafenib resistance in HCC cells, we conducted further studies. Previous reports have shown that sterol regulatory element-binding protein (SREBP)-2-driven cholesterol synthesis promotes sorafenib resistance by activating the SHH signaling pathway (30). In addition, increased cholesterol synthesis has been reported to activate SHH signaling and thereby promote colorectal tumorigenesis (31). Other studies have also demonstrated that cholesterol can activate the SHH pathway *via* SMO (32). These findings suggest that cholesterol-mediated activation of the SHH pathway may play an important role in both tumor resistance and tumorigenesis.

**Figure 6:**
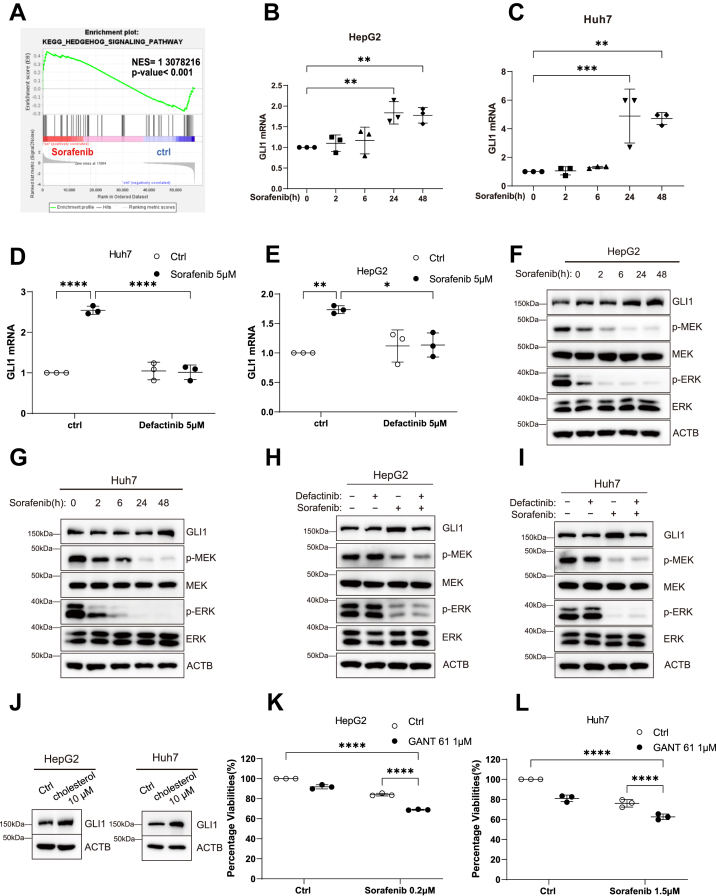
**GLI1 is a key downstream effector of cholesterol-mediated sorafenib resistance**. *A*, pathway enrichment analysis of the SHH signaling pathway in sorafenib-treated cells compared with the control group. *B* and *C*, qPCR analysis of GLI1 mRNA levels in HepG2 and Huh7 cells treated with sorafenib for 0, 2, 6, 24, and 48 h. *D* and *E*, qPCR analysis of GLI1 mRNA levels in HepG2 and Huh7 cells after 48 h of sorafenib treatment, with or without defactinib cotreatment. *F* and *G*, Western blot analysis of GLI1, p-MEK, MEK, p-ERK, and ERK protein levels in HepG2 and Huh7 cells treated with sorafenib for 0, 2, 6, 24, and 48 h. *H* and *I*, Western blot analysis of GLI1, p-MEK, MEK, p-ERK, and ERK protein levels in HepG2 and Huh7 cells after 48 h of sorafenib treatment, with or without defactinib cotreatment. *J*, Western blot analysis of GLI1 protein levels in HepG2 and Huh7 cells treated with exogenous cholesterol for 24 h. *K* and *L*, cell viability of HepG2 and Huh7 cells after 72 h of combined treatment with GANT61 and sorafenib, assessed using the Alamar Blue assay. Statistical significance for *panels A* was assessed using the GSEA algorithm (Broad Institute) with 1000 gene set permutations. Normalized enrichment score (NES) and nominal *p* value are shown. Statistical significance for *panels B and C* was determined using one-way ANOVA followed by Bonferroni’s *post hoc* test. Statistical significance for *panels D*, *E*, *K*, *and L* was determined using two-way ANOVA followed by Bonferroni’s *post hoc* test. Data are presented as mean ± SD from at least three independent experiments. Significance is indicated as Significance is indicated as *p* ≥ 0.05 (ns), *p* < 0.05 (∗), *p* < 0.01 (∗∗), *p* < 0.001 (∗∗∗), and *p* < 0.0001 (∗∗∗∗). FAK, focal adhesion kinase; GLI1, glioma-associated oncogene homolog 1.

We first analyzed RNA-Seq data and found that overall SHH pathway activity was upregulated after sorafenib treatment (Fig. 6*A*). Examination of individual pathway genes revealed that SMO and GLI1 expression was increased following sorafenib treatment but decreased in the sorafenib plus defactinib group (Fig. S3, *N*–*C*). qPCR validation confirmed that in Huh7 cells, sorafenib treatment for 24 h and 48 h upregulated SMO mRNA levels, whereas cotreatment with defactinib suppressed this effect (Fig. S3, *D* and *E*). Similarly, in both HepG2 and Huh7 cells, sorafenib treatment for 24 h and 48 h significantly increased GLI1 mRNA expression, which was effectively inhibited by defactinib (Fig. 6, *B*–*E*). At the protein level, Western blot analysis demonstrated that sorafenib treatment for 48 h markedly increased GLI1 protein levels in HepG2 and Huh7 cells (Fig. 6, *F* and *G*), whereas cotreatment with defactinib abrogated sorafenib-induced GLI1 upregulation (Fig. 6, *H* and *I*). Together, these results indicate that sorafenib upregulates the key SHH pathway effector GLI1 in HCC cells, and that this process occurs downstream of FAK signaling.

To further verify the role of cholesterol, we treated HepG2 and Huh7 cells with exogenous cholesterol and observed a similar increase in GLI1 protein expression (Fig. 6, *H* and *I*), confirming that cholesterol can indeed activate the SHH pathway and upregulate GLI1 in HCC cells. Finally, to determine whether inhibition of GLI1 could enhance the sensitivity of HCC cells to sorafenib, we applied the GLI1 inhibitor GANT61. The results showed that GANT61 in combination with sorafenib significantly suppressed the viability of both HepG2 and Huh7 cells (Fig. 6, *K* and *L*).

In summary, these findings demonstrate that sorafenib activates FAK signaling, which promotes cholesterol biosynthesis, leading to activation of the SHH pathway effector GLI1 and ultimately inducing sorafenib resistance in HCC cells.

### FAK-mediated AKT activation promotes cholesterol biosynthesis in HCC

Our previous results demonstrated that sorafenib-treated HCC cells develop resistance through FAK activation and subsequent upregulation of HMGCR, thereby promoting cholesterol biosynthesis. We further investigated the mechanism by which FAK regulates HMGCR expression. Previous studies have reported that the AKT signaling pathway, downstream of FAK, can regulate cholesterol biosynthesis *via* SREBP-2 (33, 34), and that AKT activation is widely implicated in sorafenib resistance in HCC (35, 36). Therefore, we hypothesized that AKT plays a crucial role in FAK-mediated HMGCR upregulation.

**Figure 7:**
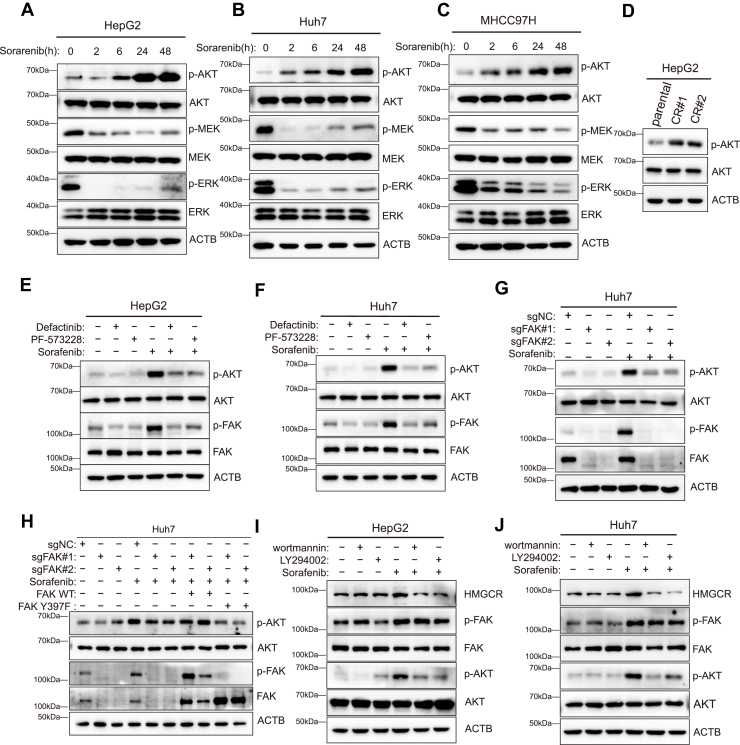
**AKT mediates the upregulation of cholesterol synthesis pathway following sorafenib treatment**. *A–C*, protein levels of p-AKT, AKT, p-MEK, MEK, p-ERK, and ERK were assessed by Western blotting following sorafenib treatment in HepG2, Huh7, and MHCC97H cells. *D*, Western blot analysis of p-AKT and total AKT protein levels in parental HepG2 cells and two sorafenib-resistant HepG2 cell lines. *E* and *F*, Western blot analysis of p-FAK, total FAK, p-AKT, and total AKT protein levels in HepG2 and Huh7 cells following cotreatment with sorafenib and a FAK inhibitor. *G*, FAK, p-FAK, p-AKT, and AKT protein levels were analyzed by Western blotting in WT and FAK-KO cells following 24-h sorafenib treatment. *H*, in FAK-KO Huh7 cells, either WT FAK or the Y397F mutant was reintroduced, followed by 24-h sorafenib treatment. HMGCR, FAK, and p-FAK protein levels were analyzed by Western blotting. *I* and *J*, p-AKT, AKT, p-FAK, and FAK protein levels were analyzed by Western blotting in HepG2 and Huh7 cells following 24-h cotreatment with the AKT inhibitor and sorafenib. FAK, focal adhesion kinase; p-FAK, phosphorylated FAK; HMGCR, 3-hydroxy-3-methylglutaryl-coenzyme A reductase.

To test this hypothesis, we first examined the effect of sorafenib treatment on AKT activation using Western blot analysis. Sorafenib treatment for 24 and 48 h markedly increased p-AKT levels in HepG2, Huh7, and MHCC97H cells (Fig. 7, *A*–*C*). Furthermore, p-AKT levels were significantly elevated in sorafenib-resistant HepG2 cell lines compared to parental cells (Fig. 7*D*). To determine whether FAK mediates AKT activation, we assessed the effect of FAK inhibition on p-AKT levels. In sorafenib-treated HepG2 and Huh7 cells, concurrent upregulation of p-FAK and p-AKT was significantly suppressed by cotreatment with defactinib or PF-573228 (Fig. 7, *E* and *F*). Moreover, in FAK-KO Huh7 cells, sorafenib no longer induced upregulation of p-AKT (Fig. 7*G*); re-expression of WT FAK restored p-AKT upregulation, whereas the phosphorylation-deficient Y397F mutant did not (Fig. 7*H*). These findings confirm that sorafenib induces AKT activation *via* FAK, and that this effect is abolished upon FAK inhibition or deletion.

Western blot analysis showed that sorafenib-induced p-FAK levels remained unchanged upon AKT inhibition, whereas HMGCR expression was significantly reduced in cells cotreated with sorafenib and the AKT inhibitor. Western blot analysis showed that, after sorafenib treatment, p-FAK levels remained unchanged despite AKT inhibition, whereas HMGCR expression was significantly reduced in the combination treatment group (Fig. 7, *I* and *J*). These results indicate that AKT acts downstream of FAK to regulate HMGCR expression.

In summary, our findings demonstrate that sorafenib-treated HCC cells activate the FAK/AKT/HMGCR signaling axis, which promotes cholesterol biosynthesis and ultimately contributes to drug resistance.

### FAK inhibitor defactinib synergistically enhances sorafenib efficacy in HCC *in vivo*

Our previous *in vitro* experiments demonstrated that cotreatment with a FAK inhibitor effectively suppressed activation of the FAK/AKT axis, reduced cholesterol biosynthesis, and overcame resistance to sorafenib. To further validate these findings, we conducted *in vivo* experiments to evaluate the antitumor efficacy of sorafenib in combination with defactinib.

**Figure 8:**
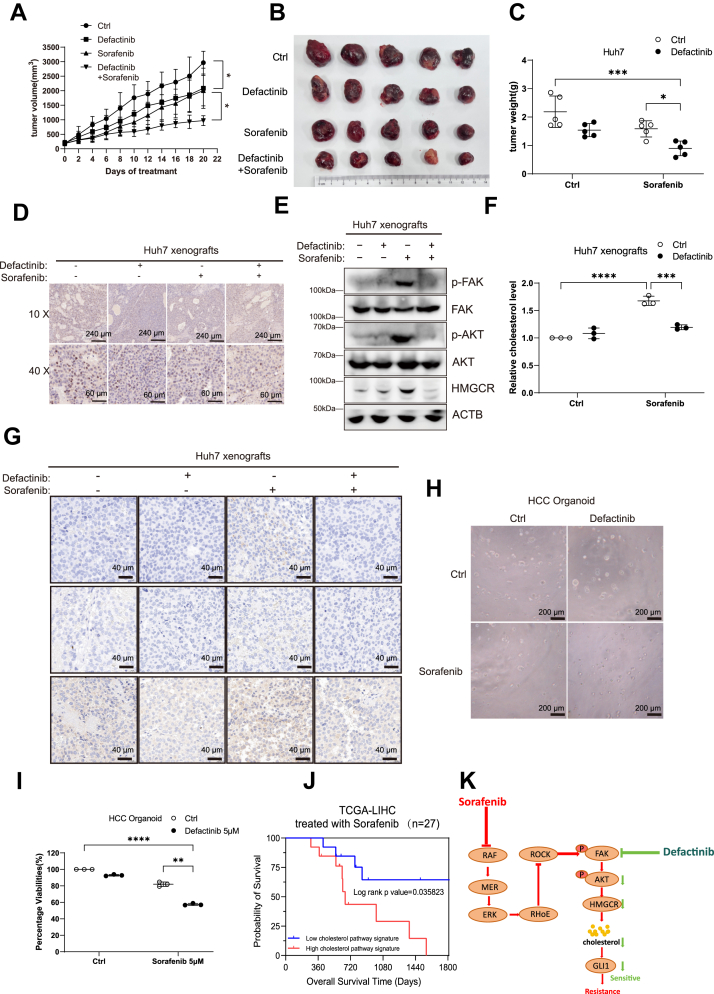
**Defactinib in combination with sorafenib inhibits tumor *in vivo***. *A–C*, Huh7 subcutaneous tumor xenograft model treated with oral defactinib (25 mg/kg/day) and sorafenib (20 mg/kg/day) starting 5 days after tumor formation. Tumor volume was measured every 2 days. On day 20, mice were euthanized, and tumors were excised, photographed, and weighed. *D*, immunohistochemical staining of tumor tissues for Ki67 expression, with brown staining indicating Ki67 positivity. Scale bars represent 240 μm for the 10 × image and 60 μm for the 40 × image. *E*, Huh7 subcutaneous tumor xenografts were treated with oral defactinib (25 mg/kg/day) and sorafenib (20 mg/kg/day) starting 5 days after tumor formation. After 7 days of treatment, tumors were harvested and analyzed by Western blotting to assess the protein expression levels of p-FAK, FAK, p-AKT, AKT, and HMGCR. *F*, measurement of total intracellular cholesterol levels in mouse tumor tissues. *G*, immunohistochemical staining of mouse tumor tissues showing protein expression of p-FAK, p-AKT, and HMGCR. *H*, morphological observation of HCC organoids after 7 days of combined treatment with defactinib and sorafenib. *I*, cell viability of HCC organoids after 7 days of combined treatment with defactinib and sorafenib, assessed using the CellTiter-Glo 3D Cell Viability Assay. *J*, Kaplan–Meier survival analysis comparing patients with high and low cholesterol pathway signature expression. Statistical significance was determined using the log-rank test. *K*, schematic model illustrating that compensatory upregulation of p-FAK mediates acquired sorafenib resistance in HCC. Sorafenib suppresses the RAF/MEK/ERK pathway while downregulating its downstream effector RhoE, thereby relieving inhibition of the RhoA/ROCK pathway and leading to increased p-FAK (Y397) levels. Activated FAK subsequently upregulates HMGCR expression through the AKT signaling pathway, enhancing cholesterol biosynthesis, which further activates the SHH pathway effector GLI1, ultimately inducing sorafenib resistance in HCC cells. In contrast, FAK inhibitors effectively block FAK activation and its downstream signaling, thereby reversing sorafenib resistance. Statistical significance for *panels A*, *C*, *F*, and *I* was determined by two-way ANOVA followed by Bonferroni’s *post hoc* test. Statistical significance for *panel J* was determined using the log-rank (Mantel–Cox) test, and the *p* value is shown in the plot. Data are presented as mean ± SD from at least three independent experiments. Significance is indicated as *p* ≥ 0.05 (ns), *p* < 0.05 (∗), *p* < 0.01 (∗∗), *p* < 0.001 (∗∗∗), and *p* < 0.0001 (∗∗∗∗). FAK, focal adhesion kinase; HMGCR, 3-hydroxy-3-methylglutaryl-coenzyme A reductase; ROCK, Rho-associated protein kinase; HCC, hepatocellular carcinoma.

We established Huh7 subcutaneous xenograft tumors in nude mice and randomly assigned them to four treatment groups: dimethyl sulfoxide, defactinib monotherapy, sorafenib monotherapy, and sorafenib+defactinib combination therapy. After 20 days of treatment, the combination therapy group exhibited significantly greater tumor growth inhibition than either monotherapy group (Fig. 8, *A* and *B*). Moreover, tumor weight was significantly lower in the combination group than in the single-agent groups (Fig. 8*C*). Ki67 staining of tumor sections revealed a markedly reduced proliferation index in the combination group, indicating reduced malignancy (Fig. 8*D*). These findings confirm that combination therapy effectively suppresses tumor growth and progression *in vivo*. Additionally, there were no significant differences in body weight or serum alanine aminotransferase and aspartate aminotransferase levels among the four groups (Fig. S4, *A*–*C*), suggesting that the combination therapy did not induce notable toxicity.

To further investigate changes in resistance-associated signaling pathways in tumor tissues, we conducted an additional *in vivo* experiment using Huh7 xenografts, in which tumors were harvested after 7 days of treatment for Western blot analysis. The results showed that, compared with the control group, sorafenib monotherapy significantly upregulated the protein levels of p-FAK, p-AKT, and HMGCR, whereas this upregulation was effectively suppressed in the combination treatment group (Fig. 8*E*). We next measured total intracellular cholesterol levels in tumor tissues and found that sorafenib monotherapy increased total cholesterol, while the combination treatment attenuated this elevation (Fig. 8*F*). Consistently, immunohistochemical staining of tumor tissues revealed markedly higher expression of p-FAK, p-AKT, and HMGCR proteins in the sorafenib monotherapy group, which was significantly inhibited in the combination group (Fig. 8*G*). These *in vivo* findings are consistent with our *in vitro* results, collectively confirming that combination treatment effectively suppresses the activation of resistance-associated signaling pathways.

Organoid models better preserve the heterogeneity and 3D architecture of primary tumors. Due to their higher clinical relevance and experimental controllability, organoids have recently emerged as valuable tools for evaluating drug combinations and serve as a useful complement to animal studies. Accordingly, we successfully established a patient-derived HCC organoid model. Drug treatment in this model demonstrated that the combination of defactinib and sorafenib synergistically inhibited organoid growth (Fig. 8*H*). Cell viability analysis using the CellTiter-Glo 3D assay further confirmed that the two agents exerted synergistic effects in suppressing the viability of HCC organoids (Fig. 8*I*). These results provide additional evidence from a biomimetic *in vitro* model that sorafenib and defactinib act synergistically to inhibit HCC progression in both *in vivo* and *in vitro* settings.

Regorafenib, a second-line therapy for HCC, also targets the RAF/MEK/ERK pathway. We therefore investigated whether regorafenib similarly induces resistance *via* p-FAK upregulation. Western blot analysis showed that treatment with regorafenib for 24 h and 48 h in HepG2, Huh7, and MHCC97H cells markedly suppressed p-MEK, p-ERK, and RhoE levels, while significantly increasing p-FAK, p-AKT, and HMGCR—effects that mirror those of sorafenib (Fig. S4, *D*–*F*). These data suggest that regorafenib may also promote resistance in HCC cells through p-FAK upregulation.

Given that sorafenib-treated HCC cells develop resistance by activating the cholesterol pathway *via* the FAK/AKT axis—and that cotreatment with sorafenib and defactinib can effectively overcome this resistance—we sought to construct a cholesterol pathway–based gene signature to predict sorafenib resistance. We first performed gene enrichment analysis on RNA-Seq data from Huh7 cells and identified 53 cholesterol pathway–related genes that were upregulated in the sorafenib monotherapy group but downregulated in the combination group (Fig. S4*G*). Among these, we selected 12 genes showing the most significant differential expression as the cholesterol pathway signature (Fig. S4*H*). Next, univariate Cox regression analysis of these genes was performed using The Cancer Genome Atlas data from 28 HCC patients treated with sorafenib. Most of these genes were found to be associated with poor prognosis (Fig. S4*I*). Kaplan–Meier survival analysis of 27 sorafenib-treated patients from The Cancer Genome Atlas showed that high expression of the 12-gene cholesterol signature correlated with significantly worse survival outcomes. These findings suggest that activation of the cholesterol biosynthesis pathway may contribute to sorafenib resistance and poor prognosis in this patient population (Fig. 8*J*).

In summary, defactinib combined with sorafenib effectively suppresses the FAK/AKT/HMGCR signaling axis *in vivo*, thereby overcoming resistance. Compared to sorafenib monotherapy, the combination therapy demonstrates superior antitumor efficacy without significant toxicity. Furthermore, the cholesterol pathway–based resistance signature may serve as a predictive biomarker for sorafenib resistance in clinical practice and inform the development of personalized therapeutic strategies for HCC.

## Discussion

Our study demonstrates that sorafenib treatment inhibits RhoE expression, thereby activating FAK in HCC cells, which leads to enhanced cholesterol biosynthesis and subsequent drug resistance. Specifically, p-FAK activates AKT, which in turn upregulates HMGCR expression to promote cholesterol biosynthesis, ultimately leading to GLI1 upregulation through the SHH pathway. Inhibition of adaptively activated FAK, along with its downstream targets AKT and cholesterol biosynthesis, using the FAK inhibitor defactinib, effectively reverses sorafenib resistance in HCC cells (Fig. 8*H*).

The role of FAK in the progression and therapeutic resistance of HCC, particularly in primary resistance, has been previously investigated. Earlier studies have shown that FAK deficiency significantly suppresses MET/CAT-driven hepatocarcinogenesis (37), In DUSP22-deficient tumors, FAK activation promotes the progression of nonalcoholic steatohepatitis–associated HCC. Additionally, FAK has been implicated in mediating primary resistance to sorafenib; its activation enhances the Wnt/β-catenin signaling pathway, thereby promoting tumor stemness, recurrence, and therapeutic resistance (38). Other studies have reported that FAK activation *via* BARMR1 induces stem-like characteristics, contributing to sorafenib resistance in HCC (39). In this study, we demonstrate that HCC cells acquire adaptive resistance to sorafenib through FAK activation. Regarding the regulatory mechanisms of FAK activation, existing evidence suggests a potential link to inhibition of the RAS/RAF/MEK pathway, although the precise mechanisms remain incompletely understood. Some studies have shown that FAK signaling is negatively regulated by the RAS/RAF/MEK cascade (40), while others have reported that FAK activation induced by RAF inhibitors can occur independently of ERK reactivation (41). Moreover, in RAF inhibitor–treated melanoma cells, FAK activation may involve the c-Jun/extracellular matrix signaling axis (42). Recent studies have revealed that, in melanoma, the Raf/MEK/ERK inhibitor avutometinib downregulates RhoE, thereby relieving its inhibitory effect on the ROCK pathway, leading to activation of the RhoA/ROCK axis and subsequent upregulation of p-FAK, ultimately contributing to drug resistance (29). Notably, combination treatment with avutometinib and a FAK inhibitor effectively overcomes this resistance mechanism and has been approved for clinical use based on results from a phase III clinical trial. In our study, we similarly found that sorafenib suppresses RhoE, thereby relieving its inhibition of the RhoA/ROCK pathway and upregulating p-FAK in HCC cells. A similar phenomenon was observed with another multikinase inhibitor, regorafenib. These findings suggest that RAS/RAF/MEK pathway inhibitors may commonly induce compensatory upregulation of p-FAK *via* RhoE suppression, ultimately leading to resistance. Thus, cotargeting FAK may represent an effective strategy to overcome this class of resistance. Given that FAK activation is a well-established driver of therapeutic resistance, combination strategies involving FAK inhibitors have demonstrated promising efficacy in multiple preclinical studies. Our findings further confirm that FAK inhibition effectively reverses sorafenib resistance and enhances its cytotoxic effects on HCC cells and tumors. Elucidating the molecular mechanisms underlying drug-induced FAK activation not only deepens our understanding of resistance development but also provides a strong theoretical foundation for designing new combination therapies, offering valuable strategies to address the clinical challenge of drug resistance.

Cholesterol has been reported to be involved in sorafenib resistance (43). One study demonstrated that hypercholesterolemic patients exhibit overexpression of the cholesterol sensor SREBP cleavage-activating protein, and that inhibition of SREBP cleavage-activating protein enhances the sensitivity of HCC cells to sorafenib (44). In our study, we similarly observed that activation of the cholesterol biosynthesis pathway following sorafenib treatment promotes resistance, and that supplementation with exogenous cholesterol can rescue the survival of HCC cells under dual-drug inhibitory conditions. Regarding the mechanisms by which cholesterol induces resistance, it has been reported that CSN6 overexpression in HCC stabilizes HMGCS1, thereby activating YAP1 signaling and promoting tumor progression (45). Another study revealed that sorafenib-treated HCC cells upregulate cholesterol biosynthesis *via* caspase-3–dependent and SREBP 2-mediated mechanisms, leading to activation of the SHH signaling pathway and drug resistance (30). Consistent with these findings, our study showed that increased cholesterol in sorafenib-treated HCC cells promoted sorafenib resistance through upregulation of GLI1 in the SHH pathway. Furthermore, our data establish cholesterol metabolism as a critical determinant of sorafenib resistance and identify FAK/AKT signaling as a key upstream driver of cholesterol biosynthesis pathway activation. Furthermore, we successfully developed a cholesterol biosynthesis–based gene signature that identifies HCC patients with high cholesterol pathway activity who may be resistant to sorafenib. For these patients, combination therapy with defactinib and sorafenib may serve as an effective therapeutic strategy, offering a new perspective and potential molecular target for precision medicine in HCC treatment.

In conclusion, FAK is a key molecular driver of sorafenib resistance in HCC. Sorafenib treatment suppresses RhoE expression, thereby relieving its inhibitory effect on the RhoA/ROCK pathway, which promotes phosphorylation of FAK at tyrosine 397 and activates the AKT-mediated cholesterol biosynthesis pathway. The resulting cholesterol subsequently activates the SHH pathway and upregulates GLI1, ultimately leading to adaptive sorafenib resistance in HCC cells. Targeting FAK with the inhibitor defactinib effectively suppresses this signaling cascade, reduces cholesterol synthesis, and restores sorafenib sensitivity. Therefore, combining defactinib with sorafenib has the potential to significantly enhance therapeutic efficacy in HCC patients, providing a promising strategy to overcome resistance and improve clinical outcomes.

## Experimental procedures

### Cell lines and reagents

HepG2, Huh7, and MHCC97H cell lines were maintained under standard laboratory conditions and cultured in Dulbecco’s modified Eagle medium (DMEM, Servicebio) supplemented with 10% fetal bovine serum (Cell-Box). Cells were incubated at 37 °C in a humidified atmosphere with 5% CO_2_ to ensure optimal growth conditions. All three cell lines (HepG2, Huh7, and MHCC97H) were authenticated by short tandem repeat profiling.

To knock out FAK in Huh7 cells, we designed two LentiCRISPRv2 plasmids targeting the following sequences: sequence 1: GTATTCAAACAGTGAAGACA and sequence 2: ATGCTACAACTAAAAATAGC. Lentivirus production was carried out by cotransfecting these plasmids with the packaging plasmids pVSVg and psPAX2 into HEK293T cells. After viral collection and infection, Huh7 cells were transduced and subsequently subjected to selection. The efficiency of FAK knockout was validated using Western blot analysis.

The sorafenib-resistant HepG2 cell line was established by gradual exposure to increasing concentrations of sorafenib. HepG2 cells were continuously treated with escalating doses of sorafenib, and IC_50_ values were monitored throughout the selection process. The treatment was continued until a stable resistant phenotype was achieved, characterized by a significantly increased IC_50_ compared to parental HepG2 cells.

Defactinib (HY-12289), PF-573228 (HY-10461), sorafenib (HY-10201), lovastatin (HY-N0504), and simvastatin (HY-17502) were obtained from MedChemExpress. Y-27632 (T1870), LY294002 (T2008), wortmannin (T6283), GANT61 (T3070), and regorafenib (T1792) were purchased from Top Science. Lipofectamine 3000 (L3000015) and DNase I (18047019) was purchased from Thermo Fisher Scientific. The siRNAs targeting RhoE and RhoA were purchased from Tsingke Biotechnology. The total cholesterol content detection reagent kit (BC1985) was obtained from Sorabio. Aspartate aminotransferase assay kits (C009-2-1) and alanine aminotransferase assay kits (C010-2-1) were purchased from the Nanjing Jiancheng Bioengineering Institute. Alamar Blue (40202ES80) was obtained from YEASEN, and Pierce 660 nm Protein Assay Reagent (22660) was purchased from Thermo Fisher Scientific. CellTiter-Glo 3D Cell Viability Assay was obtained from Promega. ABW Matrigengel (080724) was purchased from ABW, the HCC Organoid Kit (serum-free) (K2105-HCC) was obtained from bioGenous.

FAK (#3285), p-FAK (8556), p-MEK1/2 (9154), p-ERK1/2 (4370), and p-AKT (4060) antibodies were obtained from Cell Signaling Technology. AKT (10176-2-AP) MEK1/2(11049-1-AP), GLI1(66905-1-Ig), ERK1/2(11257-1-AP), HMGCR (# 13533-1-AP), and RND3 (RhoE) (66228-1-Ig) antibodies were purchased from Proteintech. ACTB (A5441) and HMGCR (A1633) antibodies were obtained from Sigma-Aldrich and Abclonal, respectively.

### Cell viability and colony formation assays

Cells were seeded in BIOFIL 96-well plates and treated with the respective drugs for 72 h. Following treatment, Alamar Blue dye was added to each well according to the manufacturer’s protocol, and fluorescence intensity was measured using a SparkControl Magellan microplate reader (TECAN) with an excitation wavelength of 560 nm and an emission wavelength of 590 nm.

For the colony formation assay, cells were plated in 6-well plates and treated with the indicated drugs for 14 days. Cell colonies were then fixed in 100% methanol and stained with 0.5% crystal violet in 25% methanol. Colony images were captured using the ChemiDoc Imaging System (Bio-Rad), and representative results from at least three independent experiments are presented.

### Western blotting and immunohistochemistry

For Western blot analysis, cells were lysed using NP-40 lysis buffer, and the supernatant was collected as the total protein extract. Protein concentration was determined using the Pierce 660 nm Protein Assay (Thermo Fisher Scientific). Equal amounts of protein (20 μg) were separated by 10% SDS-PAGE and transferred onto polyvinylidene fluoride membranes. After blocking with 5% skim milk in PBS, the membranes were incubated overnight at 4 °C with primary antibodies. Subsequently, the membranes were incubated with either an anti-mouse secondary antibody (1:5000, Southern Biotech, catalog number: 4030-05) or an anti-rabbit secondary antibody (1:5000, Thermo Fisher Scientific, catalog number: 1030-05). Protein expression was detected using an enhanced chemiluminescence reagent (Vazyme, catalog number: E423-02) and visualized with the ChemiDoc Imaging System (Bio-Rad).

For immunohistochemistry analysis, tumor tissues were fixed in 4% paraformaldehyde, paraffin-embedded, sectioned, and processed by Servicebio (Wuhan) for Ki67, p-FAK, p-AKT, and HMGCR staining. The Ki67 antibody (GB111499, Servicebio) and horseradish peroxidase–conjugated goat anti-rat IgG secondary antibody (GB23302, Servicebio) were used. The p-FAK (Cat# 8556) and p-AKT (Cat# 4060) antibodies were obtained from Cell Signaling Technology. The HMGCR antibody (Cat# 13533-1-AP) was purchased from Proteintech. Immunoreactivity was visualized using 3,3′-diaminobenzidine substrate, followed by hematoxylin counterstaining.

### Quantitative real-time PCR

Total RNA was extracted using TRIzol reagent (Vazyme) following the manufacturer’s instructions. complementary DNA synthesis was performed using the High-Capacity complementary DNA Reverse Transcription Kit (Vazyme). PCR primers were synthesized by Tsingke Biotech.

Quantitative real-time PCR was conducted in a 20 μl reaction volume using SYBR Green Pre-mix (Vazyme) on the LightCycler 96 real-time fluorescence quantitative PCR system (Roche). The thermal cycling conditions included an initial denaturation at 95 °C for 15 min, followed by 40 cycles consisting of denaturation at 95 °C for 10 s and annealing/extension at 60 °C for 30 s. Each experiment was conducted in at least three independent replicates. Gene expression levels were normalized to ACTB (β-actin) mRNA expression, and relative quantification was performed using the ΔΔCt method.

The primer sequences used for quantitative real-time PCR were as follows: HMGCR (forward: 5′-GACGTGAACCTATGCTGGTCAG-3′, reverse: 5′-GGTATCTGTTTCAGCCACTAAGG-3′); HMGCS1 (forward: 5′-AAGTCACACAAGATGCTACACCG-3′, reverse: 5′-TCAGCGAAGACATCTGGTGCCA-3′); NSDHL (forward: 5′-CAGTTTTCCACTGTGCGTCACC-3′, reverse: 5′-ACGCCCTCAAAGATGACACTGG-3′); FDPS (forward: 5′-CTTTCTTCCTGGTGGCAGATGAC-3′, reverse: 5′-AGAGCTTCAGCAGGCGGTAGAT-3′); SQLE (forward: 5′-CTCCAAGTTCAGGAAAAGCCTGG-3′, reverse: 5′-GAGAACTGGACTCGGGTTAGCT-3′); RND3 (forward: 5′-AATCACAGGCAGACGCCAGTGT-3′, reverse: 5′-CCGACTGTAAAGCTGAGCATTCG-3′); DGAT2 (forward: 5′-GCTACAGGTCATCTCAGTGCTC-3′, reverse: 5′-GTGAAGTAGAGCACAGCGATGAG-3′); LPIN1 (forward: 5′-TGCCAGTGTAGTCCAGACAGCA-3′, reverse: 5′-GTGAGGTCATCCAAGTAGACGC-3′); MOGAT3 (forward: 5′-AGCACTGCCTTACGCTCCAGAA-3′, reverse: 5′-AGGAGCCTGTGGCAAAAGCCTT-3′); FASN (forward: 5′-TTCTACGGCTCCACGCTCTTCC-3′, reverse: 5′-GAAGAGTCTTCGTCAGCCAGGA-3′); ACLY (forward: 5′-GCTCTGCCTATGACAGCACCAT-3′, reverse: 5′-GTCCGATGATGGTCACTCCCTT-3′); SLC25A20 (forward: 5′-ACCGAGTTTGCCTGGACAACCT-3′, reverse: 5′-CCCAAAGAAGCACACGGCAAAC-3′); GLI1 (forward: 5′-AGCCTTCAGCAATGCCAGTGAC-3′, reverse: 5′-GTCAGGACCATGCACTGTCTTG-3′); SMO (forward: 5′-TGCTCATCGTGGGAGGCTACTT-3′, reverse: 5′-ATCTTGCTGGCAGCCTTCTCAC-3′).

### siRNA-mediated protein knockdown

Cells were transfected at approximately 60% confluence using siRNAs targeting the protein of interest and a nontargeting control siRNA. Transfections were performed with Lipofectamine 3000 (Invitrogen) according to the manufacturer’s instructions at a final siRNA concentration of 50 nM. siRNA–lipid complexes were formed at room temperature for 15 min and then gently added dropwise to antibiotic-free medium. After 6 to 8 h, the medium was replaced with fresh complete medium, and cells were harvested 48 to 72 h posttransfection for subsequent assays. Knockdown efficiency was evaluated by Western blot analysis of the target protein.

The siRNA target sequences were as follows: siRhoE#1: CUACAGUGUUUGAGAAUUAUU; siRhoE#2: GCGGACAGAUGUUAGUACAUU; siRhoA#1: CAGAUACCGAUGUUAUACU; and siRhoA#2: GACCAAAGAUGGAGUGAGA.

### Total cholesterol measurement

Cells treated with the indicated drugs were lysed by ultrasonication in isopropanol. Following centrifugation, the supernatant was collected, and reagents were added according to the manufacturer’s instructions. The mixture was then incubated at 37 °C for 30 min, and absorbance was measured at 500 nm using a SparkControl Magellan microplate reader (TECAN). The total cholesterol content was quantified using a standard curve.

### *In vivo* xenograft model and treatment

Huh7 cells (3 × 10^6^) were subcutaneously injected into the axillary region of 4- to 6-week-old female nude mice. After 1 week, once the subcutaneous tumors reached a size of 100 to 200 mm^3^, the mice were randomly assigned to designated treatment groups and received oral drug administration. The treatment regimen included defactinib (25 mg/kg/day) and sorafenib (20 mg/kg/day). Tumor size was measured every 2 days, and tumor volume was calculated using the formula: volume = 0.5 × length × width × width. After 20 days of treatment, the mice were euthanized, and tumors were excised, photographed, and weighed. Tumor samples were collected for Ki67, p-FAK, p-AKT, and HMGCR immunohistochemical analysis. Additionally, mouse serum was collected, and alanine aminotransferase and aspartate aminotransferase levels were measured following the manufacturer’s instructions to assess potential toxicity.

### Organoid establishment and drug testing

Tumor tissue samples were washed, minced, and resuspended in 800 μl DMEM supplemented with 10% penicillin–streptomycin, followed by the addition of 200 μl dissociation solution and 10 μl DNase I. The mixture was incubated at 37 °C for 5 min, passed through a 100 μm cell strainer, rinsed with DMEM 2 to 3 times, and centrifuged at 500*g* for 5 min to collect the cell pellet. The pellet was further dissociated using a single-cell dissociation device (DocSense, Cat# SC-L1; program P3, 1–2 s pulses, 3–5 cycles). If red blood cells (RBCs) were present, 1 to 2 ml RBC lysis buffer was added and incubated at 37 °C for 3 to 5 min to lyse RBCs. The cells were then centrifuged again at 500*g* for 5 min, resuspended in Matrigel, and seeded into 24-well plates (30 μl/well). Plates were inverted and incubated at 37 °C for at least 30 min to allow Matrigel polymerization, after which 500 μl of organoid culture medium was added to each well. Organoid growth was monitored under a microscope.

For drug treatment experiments, established organoids were digested and mixed with Matrigel, and 5 μl of the mixture was seeded into each well of a 96-well plate. After polymerization at 37 °C for 30 min, 200 μl medium was added. Once organoids had recovered to their typical growth size, drug treatments were applied. After 7 days, organoid size was recorded by bright-field imaging, and cell viability was assessed using the CellTiter-Glo 3D Cell Viability Assay (Promega) according to the manufacturer’s instructions.

### Identification of therapeutic target

#### Genomic copy number variation analysis

To identify genomic loci with significant amplifications or deletions in The Cancer Genome Atlas liver hepatocellular carcinoma (LIHC) samples, we utilized GISTIC2.0 (46). A G-score was assigned based on both the amplitude of the aberration and the frequency of its occurrence across samples. Genomic regions with false discovery rate (FDR) q-values < 0.1 were considered statistically significant. Somatic copy number variation analysis was conducted using the GenePattern platform (47), developed by the Broad Institute (http://software.broadinstitute.org/cancer/software/genepattern).

#### Differentially expressed gene analysis

Differentially expressed genes were identified using DESeq2, an R package for differential expression analysis of count data. Genes with a fold change < −1 or > 1 and a q-value <0.05 were considered statistically significant.

#### Enrichment analysis

Gene set enrichment analysis (48) was performed to compare sorafenib nonresponders and responders from the phase 3 STORM trial, using C1 gene sets as the background. A total of 21 genomic loci with a Normalized Enrichment Score ≥1.25 were identified as genomic loci associated with sorafenib resistance. Additionally, 28 core-enriched genes located in the 8q24 region were identified as leading-edge genes.

To identify potential therapeutic targets, the Tier 1 druggable genome dataset (49) was intersected with the 8q24 leading-edge genes. Functional enrichment analysis was performed using the clusterProfiler package (50) to annotate Gene Ontology terms and Kyoto Encyclopedia of Genes and Genomes pathways. The enrich Gene Ontology and enrich Kyoto Encyclopedia of Genes and Genomes functions were used to conduct enrichment tests based on a hypergeometric distribution.

Gene set enrichment analysis was conducted using the clusterProfiler package, and fold changes were calculated. The gene list was ranked based on the absolute log2 fold change (|log_2_FC|) to prioritize genes for enrichment analysis.

#### CCLE database analysis

The CCLE database (51) (https://sites.broadinstitute.org/ccle) is a comprehensive public resource containing genomic, transcriptomic, and pharmacological data from thousands of cancer cell lines. To investigate the association between sorafenib sensitivity and *PTK2* expression in HCC cell lines, we downloaded sorafenib drug sensitivity data and *PTK2* expression levels from the CCLE database and analyzed their correlation.

### RNA sequencing

Total RNA was extracted using the TRIzol Reagent (Thermo Fisher Scientific) according to the manufacturer’s protocol. RNA-Seq was performed using 150-base pair paired-end reads (PE150), generating approximately 40 to 50 million reads per sample.

Raw sequencing reads were subjected to quality control filtering using Fastp and aligned to the reference genome using STAR. Gene expression levels were quantified, and differential gene expression analysis was conducted using DESeq2 (52). Functional enrichment and pathway analyses were performed using gene set enrichment analysis (48).

### Cholesterol pathway signature generation

To identify key cholesterol-related pathways, gene set enrichment analysis was performed by calculating the rank metric scores of all genes in the indicated comparisons, followed by Z-score normalization. Cholesterol pathway–related genes were selected from three curated pathways: “REACTOME_CHOLESTEROL_BIOSYNTHESIS,” “REACTOME_REGULATION_OF_CHOLESTEROL_BIOSYNTHESIS_BY_SREBP_SREBF,” and “WP_CHOLESTEROL_BIOSYNTHESIS_PATHWAY_IN_HEPATOCYTES.”

For The Cancer Genome Atlas LIHC patients treated with sorafenib, *p* values for each cholesterol pathway gene were calculated using a two-way log-rank test. The cut-off hazard ratio for defining the cholesterol pathway signature was set at 1.3. The cholesterol pathway signature score was generated using principal component analysis in 28 The Cancer Genome Atlas LIHC patients who received sorafenib treatment.

### Survival analysis

Forest plots and Kaplan–Meier survival curves were used to visualize the survival analysis results. Patients lacking survival data were excluded from the analysis. The Cox proportional hazards regression model was employed to estimate hazard ratios, *p* values, and 95% confidence intervals. Statistical significance was determined using the log-rank test.

### Patient data

This study included a total of 67 HCC patients who received sorafenib treatment in the phase 3 STORM trial, comprising 46 sorafenib nonresponders and 21 sorafenib responders (23). Additionally, 28 sorafenib-treated patients from the The Cancer Genome Atlas LIHC dataset were incorporated into the analysis. Details of the bioinformatics analyses performed are provided in the figure legends.

### Statistics

All error bars represent the mean ± SD from at least three independent experiments. Statistical analyses were performed using GraphPad Prism 9 (GraphPad Software, https://www.graphpad.com) and R v4.2.2 for data visualization and computation. For comparisons between two groups, unpaired *t*-tests were conducted. Multiple group comparisons were performed using two-way analysis of variance with Bonferroni correction. The Bonferroni-corrected two-way ANOVA was also used to assess *in vivo* combination treatment effects.

Kaplan–Meier survival curves were generated and compared using the log-rank test. Statistical methods specific to RNA-seq data are detailed in the RNA-seq methods section. Correlation analysis was conducted using Spearman’s correlation test. *p* values were adjusted using the Benjamini–Hochberg method, and FDRs were calculated. A *p* value or FDR q-value <0.05 was considered statistically significant.

## Ethics approval

All animal protocols were approved by the Animal Care and Use Committee of the College of Life Science (Wuhan University, Wuhan, China) and were in compliance with the National Research Council Guide for the Care and Use of Laboratory Animals. Human hepatocellular carcinoma tissues used for organoid generation were obtained from patients at Zhongnan Hospital of Wuhan University. All patients provided written informed consent prior to tissue collection. The study was approved by the Ethics Committee of Zhongnan Hospital of Wuhan University (approval number: KELUN2022076K) and conducted in accordance with the Declaration of Helsinki.

## Data availability

Transcriptomic, genomic, and clinical data from the The Cancer Genome Atlas LIHC studies are available in NCI Genomics Data Commons (NCI-GDC: https://gdc.cancer.gov). The transcriptomic data of 67 HCC patients that were treated with sorafenib in the phase 3 STORM trial (GSE109211) are available in the Gene Expression Omnibus database (https://www.ncbi.nlm.gov/geo/). The RNA-Seq count data can be found in Supplementary Table 1. The data generated in this study are available within the article and the supplementary data files.

## Supporting information

This article contains supporting information.

## Conflict of interest

The authors declare that they have no conflicts of interest with the contents of this article.
