# Supplementary material for: RhoE downregulation leads to enhanced cholesterol biosynthesis and sorafenib resistance in hepatocellular carcinoma
Source: J Biol Chem. 2025 Nov 11;301(12):110918. doi: 10.1016/j.jbc.2025.110918 (PMC12757643; doi:10.1016/j.jbc.2025.110918)
Supplement: Figure S1 [file mmc2.pdf]

Figure S2

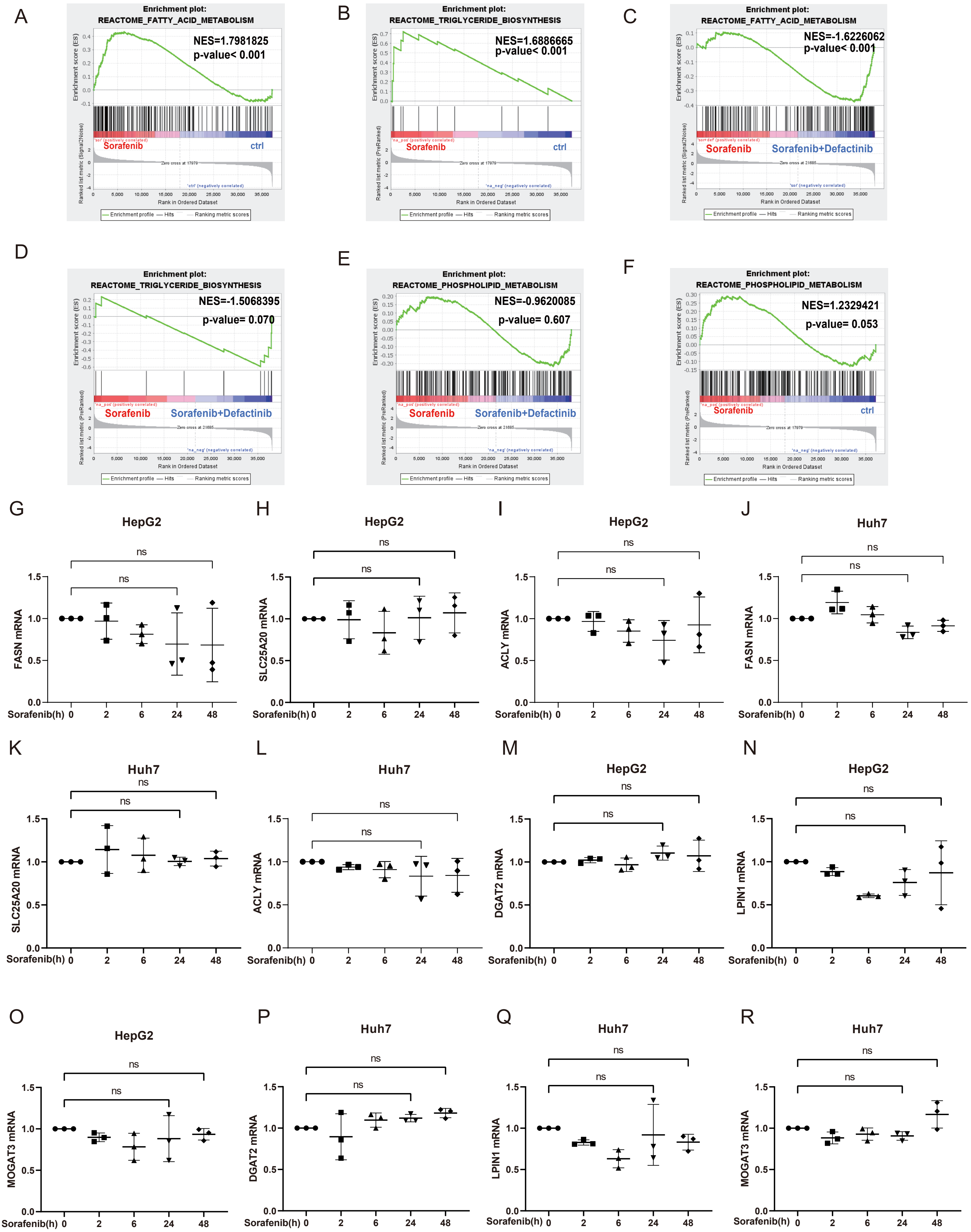
A
Enrichment plot: REACTOME_FATTY_ACID_METABOLISM
NES=1.7981825
p-value< 0.001
Sorafenib
ctrl
B
Enrichment plot: REACTOME_TRIGLYCERIDE_BIOSYNTHESIS
NES=1.6886665
p-value< 0.001
Sorafenib
ctrl
C
Enrichment plot: REACTOME_FATTY_ACID_METABOLISM
NES=-1.6226062
p-value< 0.001
Sorafenib
Sorafenib+Defactinib
D
Enrichment plot: REACTOME_TRIGLYCERIDE_BIOSYNTHESIS
NES=-1.5068395
p-value= 0.070
Sorafenib
Sorafenib+Defactinib
E
Enrichment plot: REACTOME_PHOSPHOLIPID_METABOLISM
NES=-0.9620085
p-value= 0.607
Sorafenib
Sorafenib+Defactinib
F
Enrichment plot: REACTOME_PHOSPHOLIPID_METABOLISM
NES=1.2329421
p-value= 0.053
Sorafenib
ctrl
Enrichment score (ES)
Ranked list metric
Rank in Ordered Dataset
Enrichment profile — Hits — Ranking metric scores
G HepG2 FASN mRNA
H HepG2 SLC25A20 mRNA
I HepG2 ACLY mRNA
J Huh7 FASN mRNA
K Huh7 SLC25A20 mRNA
L Huh7 ACLY mRNA
M HepG2 DGAT2 mRNA
N HepG2 LPIN1 mRNA
O HepG2 MOGAT3 mRNA
P Huh7 DGAT2 mRNA
Q Huh7 LPIN1 mRNA
R Huh7 MOGAT3 mRNA
ns
Sorafenib(h) 0 2 6 24 48
0.0 0.5 1.0 1.5

Supplementary Figure S2.

(A-F) Pathway enrichment analysis of fatty acid metabolism, triglyceride synthesis, and phospholipid metabolism in sorafenib-treated cells compared with the control group and defactinib plus sorafenib group compared with sorafenib monotherapy.

(G–L) qPCR analysis of FASN, SLC25A20, and ACLY mRNA levels in HepG2 and Huh7 cells treated with sorafenib for 0, 2, 6, 24, and 48 h.

(M–R) qPCR analysis of DGAT2, LPIN1, and MOGAT3 mRNA levels in HepG2 and Huh7 cells treated with sorafenib for 0, 2, 6, 24, and 48 h.

Statistical significance for panels A and B was assessed using the GSEA algorithm (Broad Institute) with 1,000 gene set permutations. normalized enrichment score (NES), and Nominal p-value are shown. Statistical significance for panels B–D was determined using one-way ANOVA followed by Bonferroni's post hoc test. Data are presented as mean ± SD from at least three independent experiments. Significance is indicated as 0.1234(ns), $p \geq 0.05$ (ns), $p < 0.05$ (*), $p < 0.01$ (**), $p < 0.001$ (***), and $p < 0.0001$ (****).
