# Supplementary material for: RhoE downregulation leads to enhanced cholesterol biosynthesis and sorafenib resistance in hepatocellular carcinoma
Source: J Biol Chem. 2025 Nov 11;301(12):110918. doi: 10.1016/j.jbc.2025.110918 (PMC12757643; doi:10.1016/j.jbc.2025.110918)
Supplement: Figure S2 [file mmc3.pdf]

Figure S1

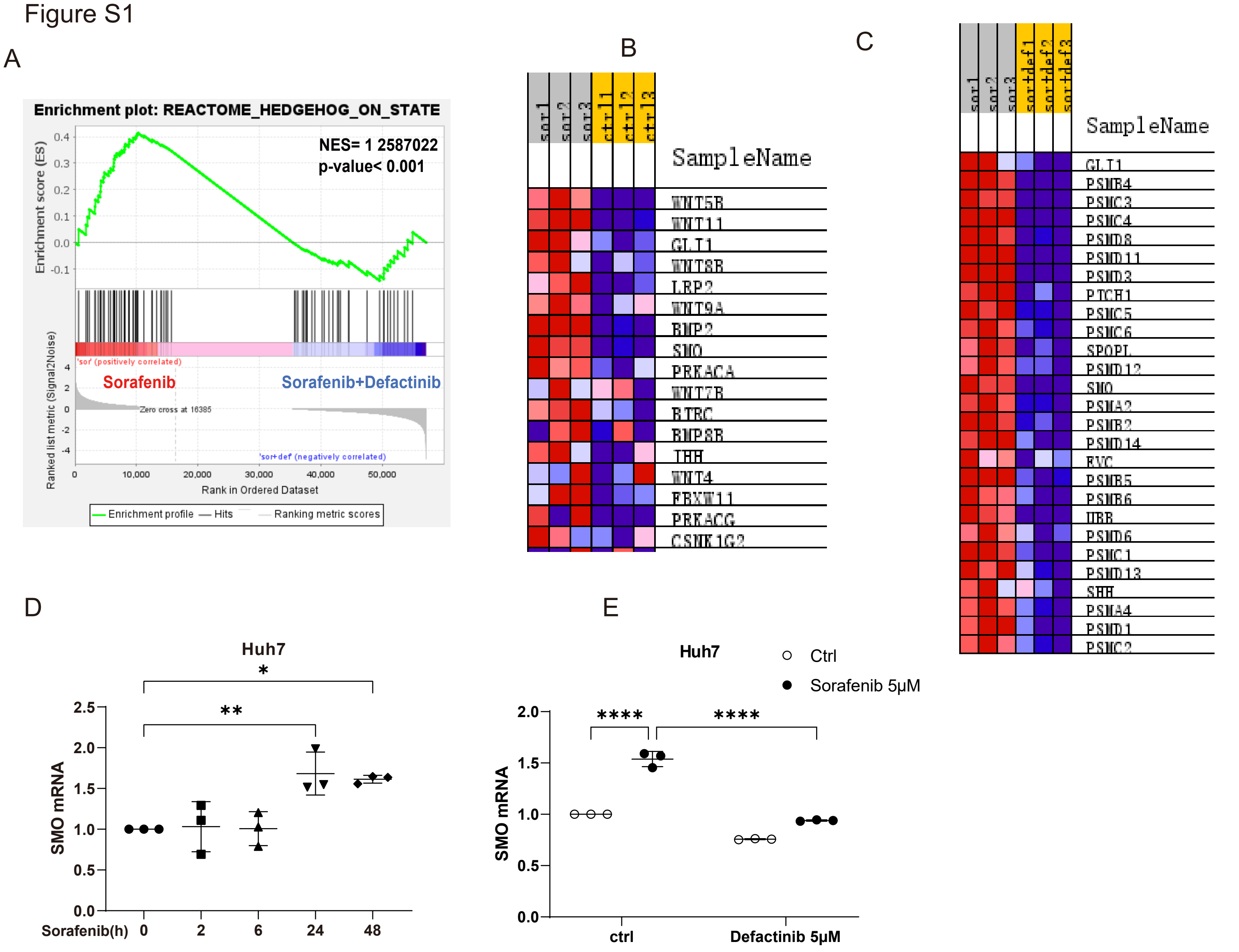

Supplementary Figure S3.

(A) Pathway enrichment analysis of the SHH signaling pathway in the defactinib plus sorafenib group compared with sorafenib monotherapy.

(B) Upregulated genes in sorafenib-treated cells compared with the control group.

(C) Downregulated genes in the defactinib plus sorafenib group compared with sorafenib monotherapy.

(D) qPCR analysis of SMO mRNA levels in Huh7 cells treated with sorafenib for 0, 2, 6, 24, and 48 h.

(E) qPCR analysis of SMO mRNA levels in Huh7 cells after 48 h of sorafenib treatment, with or without defactinib co-treatment.

Statistical significance for panels A was assessed using the GSEA algorithm (Broad Institute) with 1,000 gene set permutations. normalized enrichment score (NES), and Nominal p-value are shown. Statistical significance for panels D was determined using one-way ANOVA followed by Bonferroni's post hoc test. Statistical significance for panels E was determined using two-way ANOVA followed by Bonferroni's post hoc test. Data are presented as mean ± SD from at least three independent experiments. Significance is indicated as $p \geq 0.05$ (ns), $p < 0.05$ (*), $p < 0.01$ (**), $p < 0.001$ (***), and $p < 0.0001$ (****).
