# Supplementary material for: RhoE downregulation leads to enhanced cholesterol biosynthesis and sorafenib resistance in hepatocellular carcinoma
Source: J Biol Chem. 2025 Nov 11;301(12):110918. doi: 10.1016/j.jbc.2025.110918 (PMC12757643; doi:10.1016/j.jbc.2025.110918)
Supplement: Figure S4 [file mmc5.pdf]

Figure S4

A

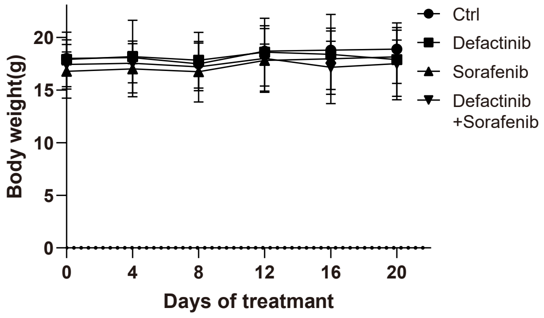

B

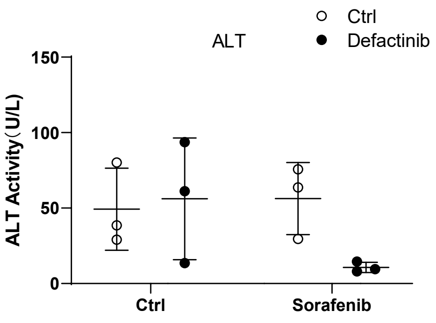

C

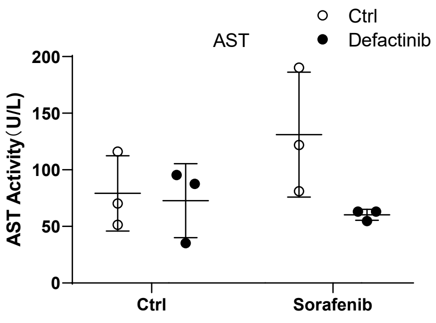

D

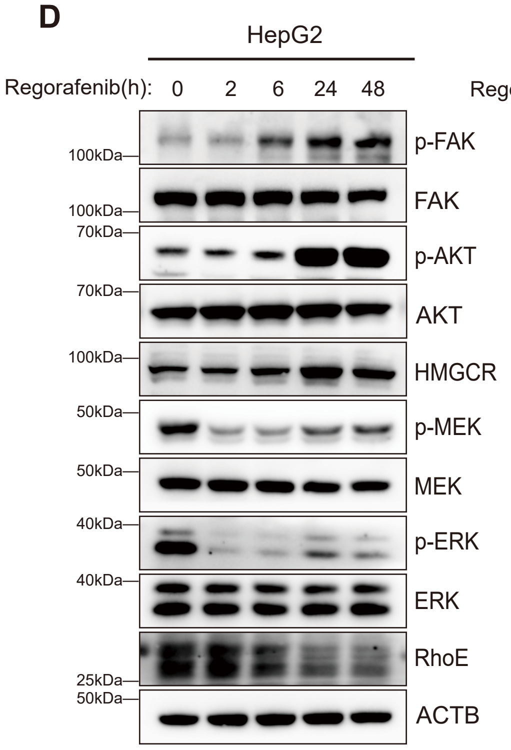

E

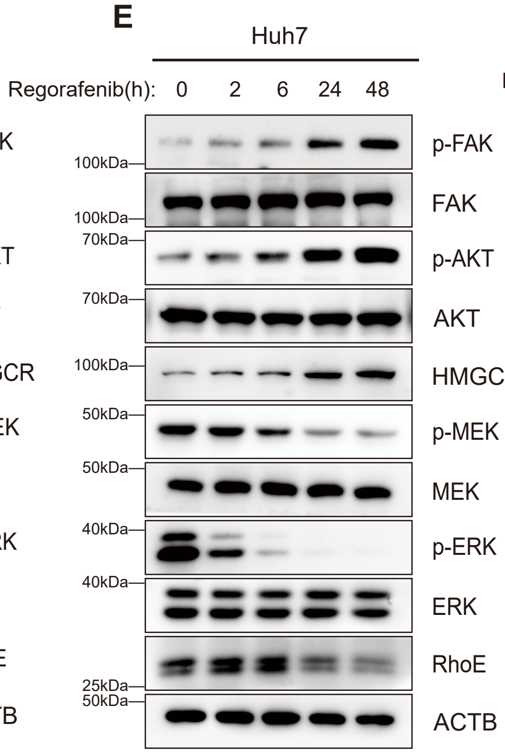

F

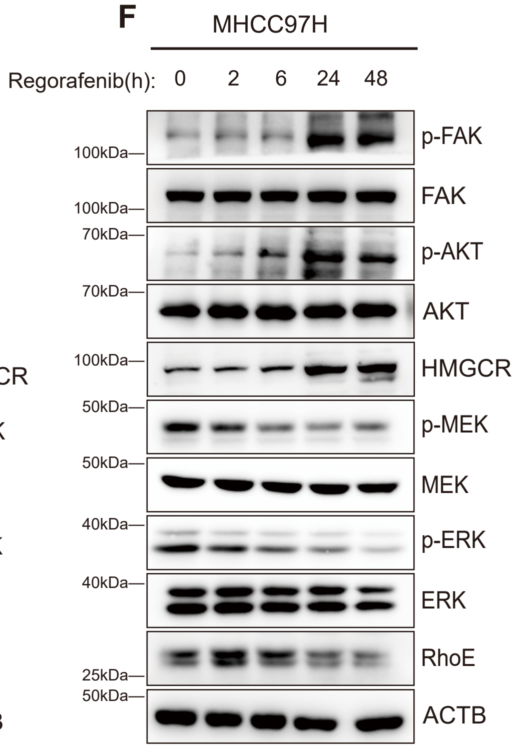

G

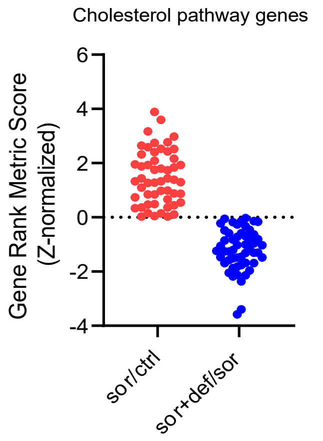

H

Cholesterol pathway signature

#	Symbol	sor/Ctrl	def+sor/sor
1	TM7SF2	3.89	-3.58
2	MVD	3.59	-2.18
3	DHCR7	2.98	-1.76
4	MVK	2.74	-0.82
5	NSDHL	2.43	-1.82
6	SQLE	1.93	-1.20
7	SCD	1.93	-2.04
8	EBP	1.88	-2.37
9	FASN	1.33	-1.68
10	FDPS	1.28	-0.72
11	ACACA	1.10	-0.70
12	PMVK	0.03	-1.48

I

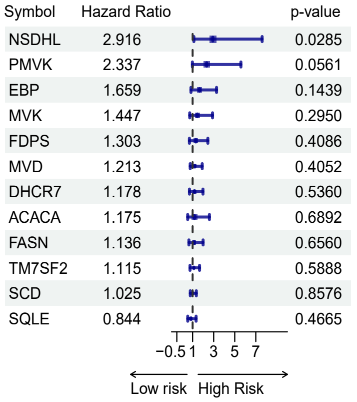

Supplementary Figure S4.

(A) Mouse body weights were monitored every 4 days during treatment to assess potential systemic toxicity.

(B, C) Serum alanine aminotransferase (ALT) and aspartate aminotransferase (AST) levels were measured to evaluate liver function and systemic toxicity after treatment.

(D–F) Protein levels of p-FAK, FAK, p-AKT, AKT, HMGCR, p-MEK, MEK, p-ERK, ERK, and RhoE were assessed by Western blotting in HepG2, Huh7, and MHCC97H cells following treatment with regorafenib.

(G, H) RNA sequencing and Gene Set Enrichment Analysis (GSEA) in Huh7 cells showed that sorafenib upregulated 53 cholesterol pathway genes (positive z-score in sorafenib vs. control), while this effect was blocked by defactinib (negative z-score in defactinib + sorafenib vs. sorafenib). A subset of 12 cholesterol-related genes was identified to define the cholesterol pathway signature.

(I) Univariate Cox regression analysis of the 12 cholesterol pathway signature genes in 28 sorafenib-treated TCGA-LIHC patients, assessing their association with clinical outcomes.

Statistical significance for panels A–C was determined by two-way ANOVA followed by Bonferroni's post hoc test. Data are presented as mean ± SD from at least three independent experiments. Significance is indicated as 0.1234(ns), $p < 0.0332$ (*), $p < 0.0021$ (**), $p < 0.0002$ (***), and $p < 0.0001$ (****). For panel H, differential expression analysis was performed using DESeq2, and significance was assessed by Wald test with Benjamini–Hochberg correction for multiple comparisons. For panel I, univariate Cox proportional hazards regression analysis was performed to evaluate the association between gene expression and overall survival, with hazard ratios (HR), 95% confidence intervals (CI), and p-values reported.
